# Identification and Verification of Five Potential Biomarkers Related to Skin and Thermal Injury Using Weighted Gene Co-Expression Network Analysis

**DOI:** 10.3389/fgene.2021.781589

**Published:** 2022-01-03

**Authors:** Ronghua Yang, Zhengguang Wang, Jiehua Li, Xiaobing Pi, Xiaoxiang Wang, Yang Xu, Yan Shi, Sitong Zhou

**Affiliations:** ^1^ Department of Burn Surgery and Skin Regeneration, The First People’s Hospital of Foshan, Foshan, China; ^2^ Department of Orthopedics, The First Affiliated Hospital of China Medical University, Shenyang, China; ^3^ Department of Dermatology, The First People’s Hospital of Foshan, Foshan, China; ^4^ Department of Molecular Pharmacology, School of Medicine, Nankai University, Tianjin, China; ^5^ Department of Wound Repair and Institute of Wound Repair, The Second Clinical Medical College, Jinan University (Shenzhen People’s Hospital), Shenzhen, China; ^6^ The First Affiliated Hospital, Jinan University, Guangzhou, China

**Keywords:** burn injury, WGCNA, skin wound, peripheral blood, ROC

## Abstract

**Background:** Burn injury is a life-threatening disease that does not have ideal biomarkers. Therefore, this study first applied weighted gene co-expression network analysis (WGCNA) and differentially expressed gene (DEG) screening methods to identify pivotal genes and diagnostic biomarkers associated with the skin burn process.

**Methods:** After obtaining transcriptomic datasets of burn patient skin and normal skin from Gene Expression Omnibus (GEO) and performing differential analysis and functional enrichment, WGCNA was used to identify hub gene modules associated with burn skin processes in the burn patient peripheral blood sample dataset and determine the correlation between modules and clinical features. Enrichment analysis was performed to identify the functions and pathways of key module genes. Differential analysis, WGCNA, protein-protein interaction analysis, and enrichment analysis were utilized to screen for hub genes. Hub genes were validated in two other GEO datasets, tested by immunohistochemistry for hub gene expression in burn patients, and receiver operating characteristic curve analysis was performed. Finally, we constructed the specific drug activity, transcription factors, and microRNA regulatory network of the five hub genes.

**Results:** A total of 1,373 DEGs in GSE8056 were obtained, and the top 5 upregulated genes were *S100A12*, *CXCL8*, *CXCL5*, *MMP3*, and *MMP1*, whereas the top 5 downregulated genes were *SCGB1D2*, *SCGB2A2*, *DCD*, *TSPAN8*, and *KRT25*. DEGs were significantly enriched in the immunity, epidermal development, and skin development processes. In WGCNA, the yellow module was identified as the most closely associated module with tissue damage during the burn process, and the five hub genes (*ANXA3*, *MCEMP1*, *MMP9*, *S100A12*, and *TCN1*) were identified as the key genes for burn injury status, which consistently showed high expression in burn patient blood samples in the GSE37069 and GSE13902 datasets. Furthermore, we verified using immunohistochemistry that these five novel hub genes were also significantly elevated in burn patient skin. In addition, MCEMP1, MMP9, and S100A12 showed perfect diagnostic performance in the receiver operating characteristic analysis.

**Conclusion:** In conclusion, we analyzed the changes in genetic processes in the skin during burns and used them to identify five potential novel diagnostic markers in blood samples from burn patients, which are important for burn patient diagnosis. In particular, MCEMP1, MMP9, and S100A12 are three key blood biomarkers that can be used to identify skin damage in burn patients.

## Introduction

Thermal injury is a challenging disease and a leading cause of death worldwide (Greenhalgh, 2019). Burns affect approximately 300 million people worldwide annually and have high morbidity and mortality rates, resulting in 176,000 people dying from burn injuries in 2015 (Haagsma et al., 2016). Moreover, severe burns can rapidly disrupt body homeostasis, leading to multi-organ dysfunction and life-threatening injuries (Auger et al., 2017). A key limiting factor for poor clinical outcomes in burn patients is the lack of reliable diagnostic tools to identify critical burn events and their extent, and subsequently initiate targeted intensive treatment (Niggemann et al., 2021). Therefore, clarifying the underlying pathogenesis of burn injury and exploring effective treatments for burn injuries are urgently required.

Burn patient prognosis is improving with the progress of modern medicine; however, due to the limitations and lags of traditional burn diagnosis, patients do not receive appropriate treatment. Research has focused on how to distinguish different degrees of burns by molecular diagnostic methods and on the application of appropriate treatment strategies in a timely manner. Burns are traditionally graded into three levels of thickness based on the degree of tissue damage: superficial (first-degree burns), partial-thickness (second-degree burns), and full-thickness (third-degree burns). Partial-thickness/second-degree burns can be further subdivided into superficial- and deep-part-thickness burns (McGill et al., 2007; Greenhalgh, 2019). Clinical diagnosis by visual and tactile examination remains the current standard for determining the depth of a patient’s burn injury. This method has a serious lag in determining the patient’s condition and does not provide timely information regarding the patient’s progress due to the rapid progression of burn injury (Lee et al., 2020). Therefore, the development of an effective molecular diagnostic burn technique is necessary to improve early burn care, reduce complications, and decrease treatment-associated costs. The inflammatory response triggered by thermal injury frequently transcends the local environment, leading to local verification and changes in blood flow. We hypothesize that the extensive perturbations of the skin caused by thermal injury lead to differential changes in gene expression in peripheral blood tissues, which can be useful for diagnosing burns and identifying changes in burn conditions.

With the rapid development of next-generation sequencing technology, numerous new computational algorithms have been developed to help identify disease-specific biomarkers. Weighted gene co-expression network analysis (WGCNA) (Langfelder and Horvath, 2008), a novel systems biology method, assists in the construction of free-scale gene co-expression networks and the detection of gene modules and hub genes. We can determine which modules are associated with disease phenotypes by analyzing the relationship between modules and clinical features (Zhu et al., 2021).

**FIGURE 1:**
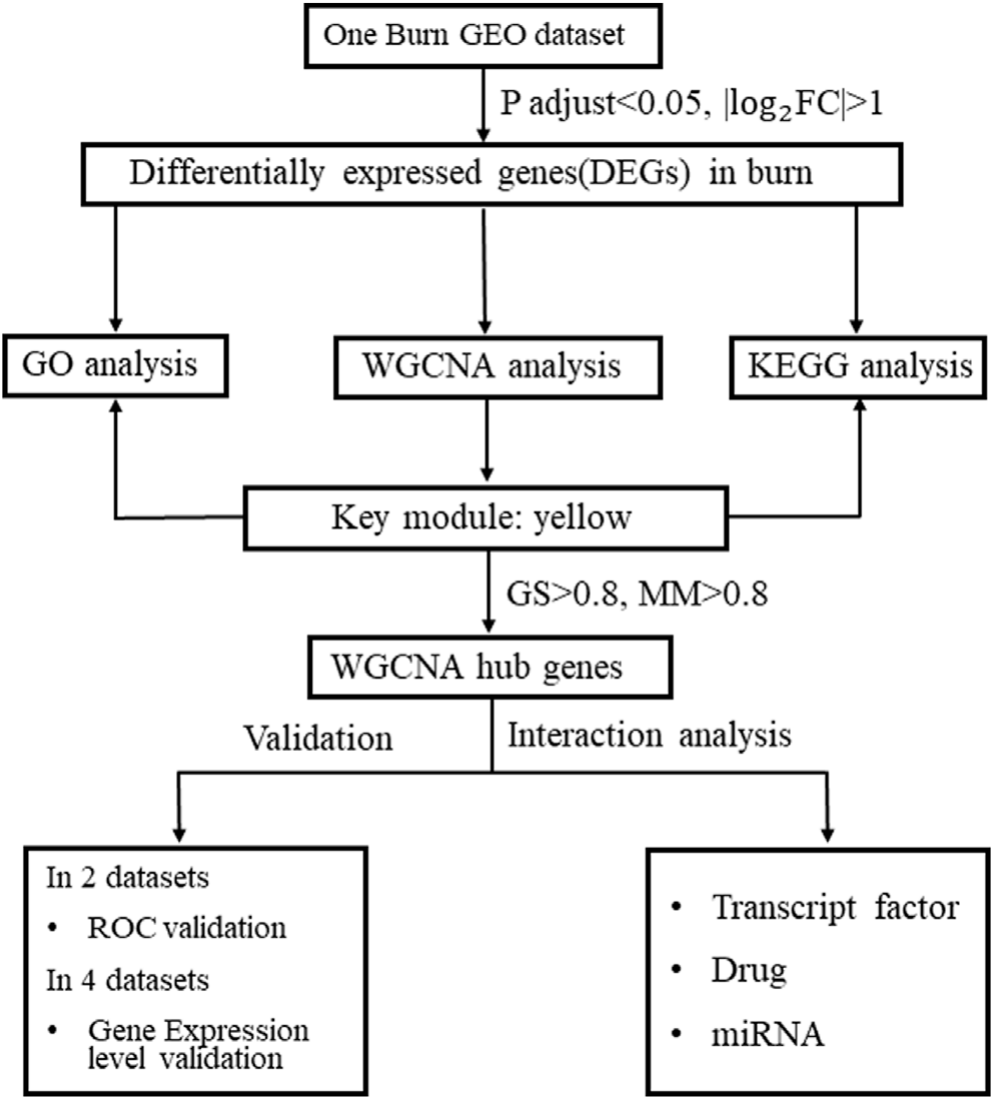
Study flowchart. (Study workflow. FC, fold change; GEO, Gene Expression Omnibus; GO, Gene Ontology; GS, gene significance; KEGG, Kyoto Encyclopedia of Genes and Genomes; MM, module membership; WGCNA, weighted gene co-expression network analysis).

In our study, we aimed to identify potential biomarkers associated with burn injuries. The secondary aim was to assess the discriminative capacity of novel mRNA biomarkers in peripheral blood for burn injury diagnosis by integrated analysis. The flowchart illustrated in the present study is presented in Figure 1. First, we identified differential expression profiles between burn and normal tissues using the GSE8056 dataset. Next, we extracted the top 100 differentially expressed genes (50 upregulated and 50 downregulated) according to log2|FC| size as the most significantly differentially expressed genes (DEGs). Next, we identified the gene module with the greatest correlation with the burn process and investigated the connectivity between this gene module and burned skin features using WGCNA. The key module with the highest level of significant correlation with burned skin injury was identified, and the genes with |gene significance| >0.8 and |module membership| >0.8 were selected as hub genes in the key yellow module. Gene Ontology (GO) enrichment and Kyoto Encyclopedia of Genes and Genomes (KEGG) pathway analyses were performed to clarify the possible functions of key modules. Furthermore, DEGs were subjected to a protein-protein interaction (PPI) network analysis to identify hub genes (degree ≥5). The overlapping genes between hub genes in the key module and hub genes in DEGs were defined as key genes (*ANXA3*, *MCEMP1*, *MMP9*, *S100A12*, and *TCN1*), and the expression levels of the five hub genes were evaluated in burn-related GEO datasets and verified by immunohistochemical (IHC) analysis of the collected skin tissues. This is the first report of utilizing WGCNA to explore skin tissue damage-related biomarkers of burn injury. This study lays the foundation for exploring the molecular mechanisms of burn injury and contributes to the identification of potential diagnostic biomarkers for burn injuries.

## Materials and Methods

### GEO Datasets

In this study, we obtained a gene expression microarray from the NCBI GEO official website (Barrett et al., 2013) (https://www.ncbi.nlm.nih.gov). The microarray data were all obtained from the GPL570 platform with the accession number GSE8056 (Ou et al., 2015) (number of burn wound tissues = 9, number of normal skin tissues = 3, tissue source: skin), GSE19743 (Zhou et al., 2010) (number of patient samples = 114, number of normal samples = 63, tissue source: blood), GSE37069 (Peterson et al., 2014) (number of patient samples = 553, number of normal samples = 37, tissue source: blood), and GPL15433 platform for RNAseq count expression profile data GSE139028 (Schutte et al., 2020) (number of patient samples = 6, number of normal samples = 3, tissue source: skin).

### DEG Analysis

Microarray data from three datasets were pre-processed using the robust multichip analysis algorithm using Affymetrix default analysis settings (Shanahan et al., 2012). In the event of multiple probes corresponding to one gene, we took the average value as the expression level of the gene. First, burn patients and healthy people (serving as controls) were analyzed for differential expression. The DEGs from GSE8056 were identified using the Limma package (Ritchie et al., 2015) in R on normalized count data. The parameters |logFC| > 2 and *p* < 0.05 were used as the screening criteria for DEGs. Next, top 100 genes (top 50 upregulated and 50 downregulated) were selected as the significantly DEGs for further analysis. Moreover, we focused on tagging the top 10 genes (5 upregulated and 5 downregulated) and plotted circles using the OmicsCircos (Hu et al., 2014) package to visualize the distribution of the most DEGs on the chromosomes and their expression in each sample. For RNAseq data in GSE139028, expression levels were transcripts per million (TPM)-normalized and ENSG-ID transformed. The TPM values were used to evaluate the gene expression levels.

### The GO Term Enrichment and Pathway Analysis of Differential Gene Expression

To evaluate the biological functions and pathways affected by differential gene sets affecting burn patients, the DEG sets from GSE8056 were used to perform GO and KEGG pathway enrichment analysis using the clusterProfiler package (Yu et al., 2012), with Q (Bonferroni-corrected *p*-value) <0.05 set as the statistically significant threshold. GO terms, including biological processes (BPs), cellular components (CCs), molecular functions (MFs), and KEGG pathways with *p* < 0.05 and false discovery rate <0.05, were considered statistically significant. The GO and KEGG analysis results were visualized using the “ggplot2” R package.

### Construction of Co-expression Modules by WGCNA of Datasets

For co-expression analysis, the DEGs from GSE8056 were selected as hub gene sets for the construction of a WGCNA (Langfelder and Horvath, 2008). We used this gene set with the expression profile dataset GSE19743 to construct the gene co-expression networks. Thereafter, the network modules correlated with the burn process and hub genes in modules were identified, and the corresponding soft thresholds were selected to filter out the key modules to achieve a scale-free network R^2^ close to 0.9. To further determine the potential biological functions and pathways in the burn injury-related module, we performed GO and KEGG pathway enrichment analyses of the target modules. Meanwhile, key gene sets were screened based on gene significance >0.2 and module membership >0.8. Then, the key gene sets were extracted for GO functional enrichment and KEGG pathway analyses.

### GO Enrichment and KEGG Pathway Analysis of Genes in Each Module

GO enrichment and KEGG pathway analyses were performed to determine the potential BPs, CCs, and MFs of genes in each module. Furthermore, significant KEGG pathway and GO enrichment analysis were implemented by the hypergeometric test, with an adjusted *p*-value (q value) < 0.05 considered significant. The GO enrichment analyses with the top 30 GO enrichment results (10 BPs, 10 CCs, and 10 MFs) were plotted for the bar graphs, whereas GO and KEGG pathway analyses were visualized using the “ggplot2” R package.

### PPI Network and the Identification of Hub Genes

To explore the PPIs between hub genes, the hub genes in the key modules filtered using WGCNA were imported into the STRING database (https://www.string-db.org/), which was continuously amplified to obtain a PPI network containing 14 nodes and 21 edges as a way to investigate the role of burn-related DEGs in the network. We identified the five most essential burn injury-associated genes based on the differential expression, functional enrichment results, and PPI network analysis results.

### Expression Analysis and Receiver Operating Characteristic Curves of the Five Hub Genes

To validate the accuracy of the obtained key genes, the accuracy of each key gene was evaluated by receiver operating characteristic (ROC) validation and the area under the curve (AUC) value. We used the five identified key genes as the validation set data using GSE37069 with GSE13902 expression data and performed ROC validation using the pROC (Robin et al., 2011) R language package to verify the classification efficacy of the key genes in the validation set data. Alternatively, to verify the accuracy of the obtained key genes, t-tests were performed to determine significant differences in gene expression levels between burn patients and normal controls. Statistical significance was set at *p* < 0.05.

### The Collection of Patient Tissue Specimens and IHC Staining

We collected 13 burn patient skin samples (deep second-degree burn tissue) from 2020 to 2021 from burn patients associated with the Chinese Han population. We received approval from the subject review committee of Foshan First People’s Hospital, and the patients signed an informed consent form and were informed prior to sample collection. After dehydration, formalin-fixed and paraffin-embedded skin tissues were sectioned at 4 μm for IHC. Antigen retrieval was performed by incubating the samples in citrate buffer (pH 6.0) for 15 min. After blocking with a mixture of methanol and 0.75% hydrogen peroxide, sections were incubated overnight with primary antibodies (ANXA3, Abcam, Cambridge, United Kingdom, 1:100; MCEMP1, Abcam 1:150; S100A12, Signalway Antibody, MD, United States, 1:100; TCN1, Invitrogen, MA, United States, 1:50; MMP9, Signalway Antibody, 1:50), followed by incubation with a secondary antibody conjugated with horseradish peroxidase (goat anti-rabbit, 1:500, Cell Signaling Technology, MA, United States). Sections were washed three times with phosphate-buffered saline and incubated with diaminobenzidine. Next, we performed a comprehensive IHC score according to the total degree of staining and the area of positive cells, and the scoring criteria and steps described in our previous article (Zhou et al., 2021).

### Analysis of Drug Activity, Transcription Factors, and microRNA Interaction Network of the Five Hub Genes

To further analyze the relationship between the action of key genes and drugs, we used three leading drug-target databases, Drugbank (Wishart et al., 2018) (https://www.drugbank.com/), DGIdb (Cotto et al., 2018) (https://dgidb.genome.wustl.edu/), and PubChem (Wang et al., 2017) (https://pubchem.ncbi.nlm.nih.gov/), to identify drugs acting on the five hub genes based on the active structural domains of drugs and to construct a network of target gene-drug interactions. For target gene transcription factor analysis, we used the ChEA3 (https://maayanlab.cloud/chea3/) prediction database (Keenan et al., 2019) to construct a network between transcription factors and genes. Additionally, the targets of experimentally validated microRNA (miRNA)-mRNA interactions were screened based on Starbase (V2.0) (Li et al., 2014), miRTarBase (Huang et al., 2020), and TarBase (Karagkouni et al., 2018). Next, the collected mutual relationship data were imported into Cytoscape (Shannon et al., 2003) for network visualization.

## Results

### Study Flowchart

The research flowchart is presented in Figure 1.

### Differentiation Analysis

**FIGURE 2:**
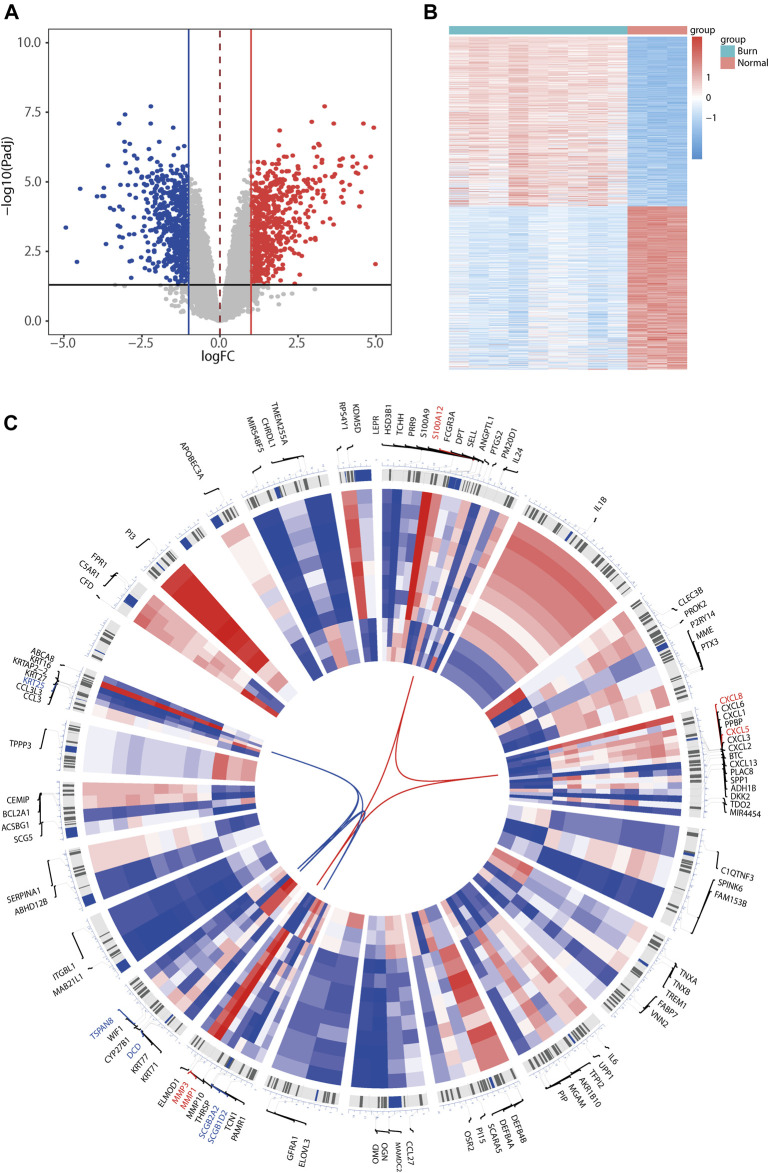
Heatmap, volcano plot, and chromosome circos plot for differentially expressed genes identified in the GSE8056 dataset. The volcano map **(A)** indicates the difference of up-and downregulated genes, where red represents upregulation and blue represents downregulation. Heatmap **(B)** represents the differentially expressed genes expression patterns of differentially expressed genes in the upper burn patients versus the normal population group. Circos plot **(C)** shows the top 100 differentially expressed genes expression patterns and the distribution of the chromosomal location where they are located, with the outer circle representing the chromosome and the location of the gene in the chromosome, and the heatmap in the inner circle representing the expression of the top 100 differentially expressed genes (DEGs) in the burn dataset GSE8056. The top 5 upregulated differentially expressed genes (red) and top 5 downregulated differentially expressed genes (blue) according to |log2FC| values are connected in red and blue in the center of the circos plot, respectively.

Differential analysis was performed using GSE8056 (burn skin samples vs. normal skin samples). |logFC (fold change)| >1 for upregulated genes and |logFC| >-1 for downregulated genes were selected as standards. A total of 1,373 DEGs (adjusted *p*-value <0.05) were obtained, of which 673 were upregulated and 700 were downregulated. Volcano and heat maps of the differentially expressed genes are plotted in Figures 2A,B, and it can be seen that the differentially expressed genes exhibit significantly different expression patterns between the burned and normal samples. Next, we extracted the top 100 differentially expressed genes (50 upregulated and 50 downregulated) as the most significant differentially expressed genes according to log2|FC| size. For a visual representation of the top 100 differentially expressed genes, the R package Omiccircos was used to draw the circos-plot for visualization (Figure 2C). Heat maps in the circos-plot were drawn according to the expression patterns in differential analysis corresponding to the samples, with upregulated and downregulated genes connected by red and blue lines, respectively. The top five upregulated genes were *S100A12*, *CXCL8*, *CXCL5*, *MMP3*, and *MMP1*, whereas the top five downregulated genes were *SCGB1D2*, *SCGB2A2*, *DCD*, *TSPAN8*, and *KRT25*.

### GO Enrichment Analysis and KEGG Signaling Pathway Analysis of DEGs in GSE8056

**FIGURE 3:**
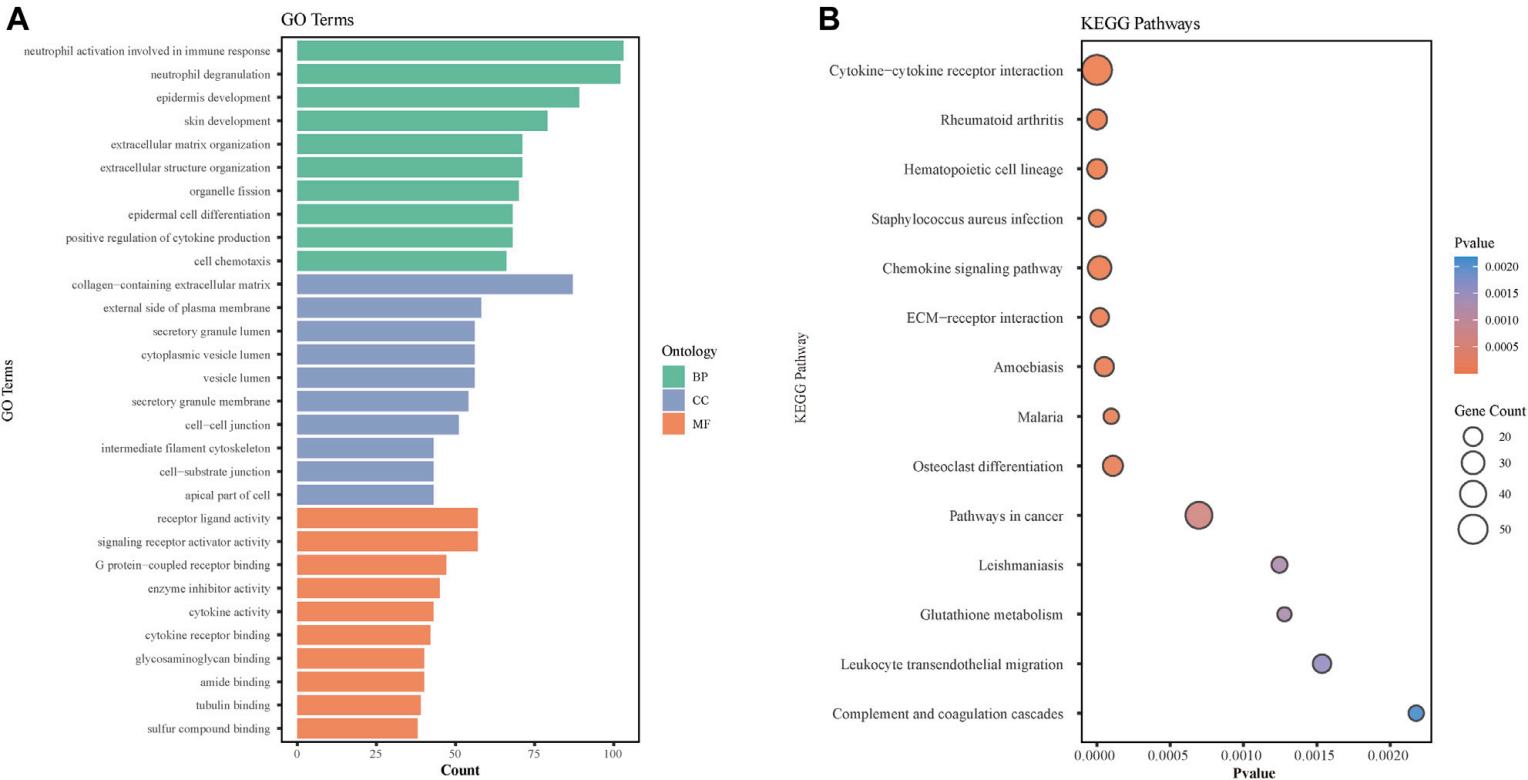
GO and KEGG pathway analyses of DEGs identified in the GSE8056 dataset. **(A)** The histogram represents the GO pathway analysis of DEGs. The green bar represents BP (Biological process), blue represents CC (Cellular Component), and orange represents MF (Molecular Function). **(B)** Bubble diagram of the Kyoto Encyclopedia of Genes and Genomes (KEGG) enrichment analysis of differential genes.

To further explore the molecular mechanism involved in the burn injury skin tissues, we performed GO enrichment and pathway enrichment analysis based on the differential gene set previously obtained from the GSE8056 database and found that GO enrichment results were significantly enriched in BPs such as immunity, epidermal development, skin development, CCs such as collagen extracellular matrix (ECM), and MFs such as signal transduction receptor activity (Figure 3A). KEGG pathway analyses of DEGs were significantly enriched for cytokine-cytokine receptor interaction, chemokine signaling pathway, ECM-receptor interaction, glutathione metabolism, and osteoclast differentiation pathways (Figure 3B).

### WGCNA and Identification of Hub Genes

**FIGURE 4:**
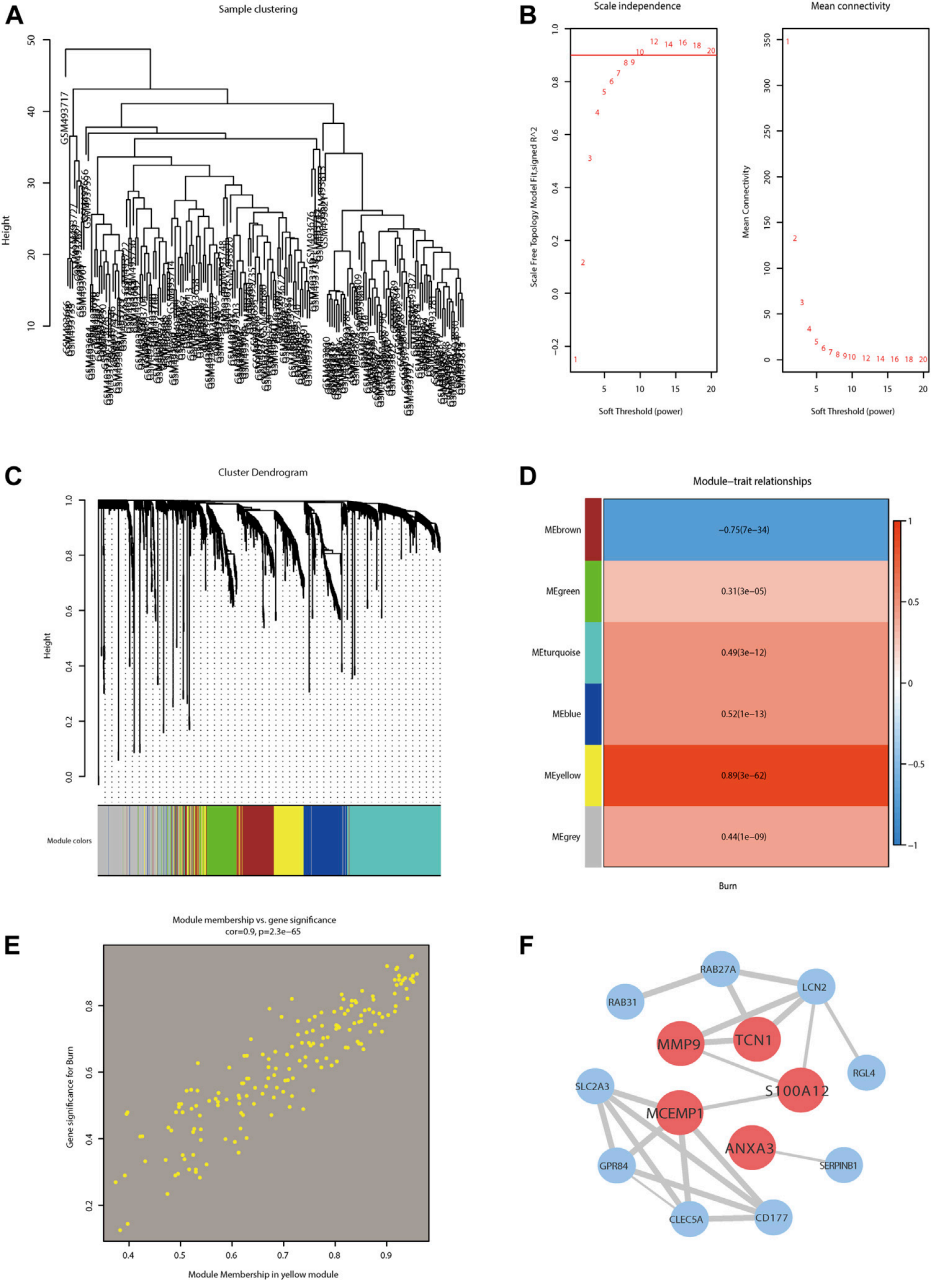
Weighted gene co-expression network analysis (WGCNA) of burn-related key modules with PPI network analysis of key modules. **(A)** Sample clustering dendrogram of GSE19743 to detect outliers. **(B)** Analysis of the scale-free fit index **(left)** and the mean connectivity **(right)** for selecting various soft-thresholding powers (β). **(C)** Clustering dendrogram for genes in burn traits; each color below represents one co-expression gene module. **(D)** Heatmap depicting correlations between module and burn traits. **(E)** Scatter plot of the key module. Each point in the scatter plot represents one gene. **(F)** Hub genes in yellow module revealed by PPI using the cytoscape software.

The sample clustering dendrogram of GSE19743 (Figure 4A) showed no obvious discrepancy between the samples incorporated into the WGCNA. We selected 8 as the optimal soft threshold power based on the scale-free topology model and the mean connectivity (Figure 4B). The gene cluster dendrogram is shown in Figure 4C, where each leaf and branch on the tree represents a gene and co-expression module, respectively. The heat map (Figure 4D) illustrates the correlation between different modules and burn traits. We obtained 5 modules except for the gray module, in which the yellow consensus module was the most relevant module with burn traits (correlation value = 0.89; significance level *p* < 0.05). Furthermore, 30 hub genes were selected in yellow co-expression modules with gene significance >0.8 and module membership >0.8. We constructed a scatter plot of the characteristic hub genes in the yellow module (Figure 4E). We then visualized the most significant module in the PPI network using the Cytoscape software (Figure 4F).

### GO Enrichment and KEGG Signaling Pathway Analyses of Differentially Expressed Genes in Different Modules

**FIGURE 5:**
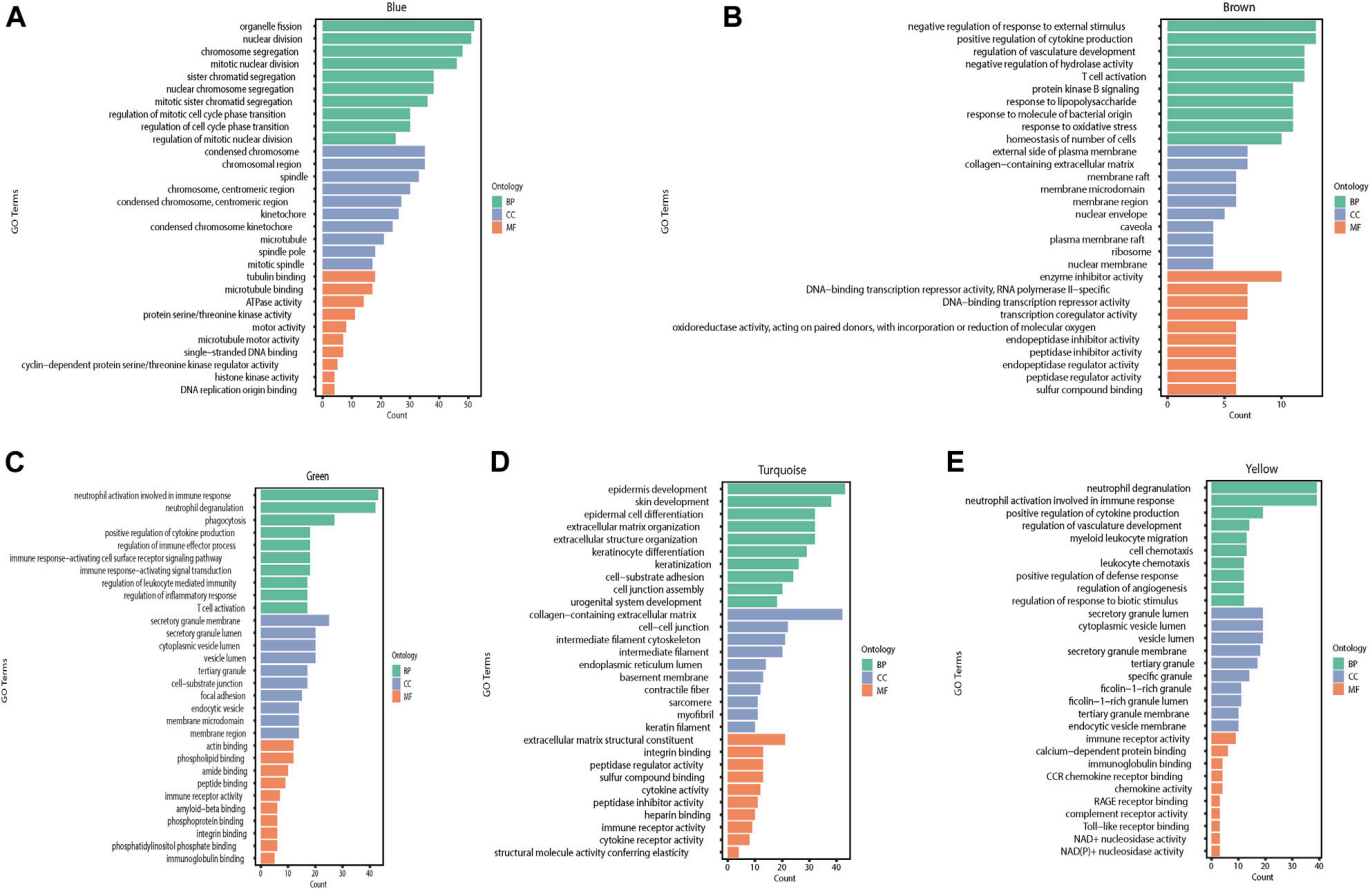
The GO enrichment results of different modules in WGCNA are shown in the histogram, with different colors representing different categories of genes. Green represents BP, blue represents CC, and orange represents MF (BP: Biological process, CC: Cellular component, MF: Molecular function). **(A)** GO enrichment analysis results of the blue module. **(B)** GO enrichment analysis results of the brown module. **(C)** GO enrichment analysis results of the green module. **(D)** GO enrichment analysis results of the turquoise module. **(E)** GO enrichment analysis results of the yellow module.

**FIGURE 6:**
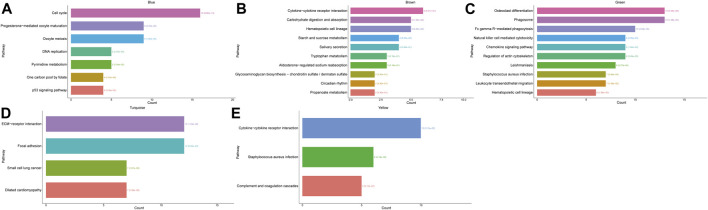
Histogram of KEGG enrichment results of the modules with the count value and significant *p*-value. **(A)** Results of KEGG enrichment analysis for the blue module. **(B)** Results of KEGG enrichment analysis for the brown module. **(C)** Results of KEGG enrichment analysis for the green module. **(D)** Results of KEGG enrichment analysis for the turquoise module. **(E)** Results of KEGG enrichment analysis for the yellow module.

We performed GO and KEGG pathway enrichment analyses for each module separately to identify the BPs, CCs, and MFs affected by each module in the burn injury process. We used the statistical method of hypergeometric test and selected *p*-values < 0.05 with corresponding Q-values < 0.05 as the significantly enriched pathways and MFs and found that the blue module was mainly correlated with mitotic nuclear division and chromosome segregation, and affects MFs such as microtubules and microfilaments of cells. In the brown module, the genes primarily affected a series of pathways and MFs at the level of signal regulation, such as protein kinase B signaling and negative regulation of response to external stimuli. The main function of the green module is to affect immune-related BPs, such as neutrophil degranulation and phagocytosis. The most significant BPs for the turquoise module are epidermal development and skin development. Finally, the yellow module plays an important role in the immune response of the body, emergency response to inflammation, and epidermal development (Figures 5, 6).

We performed KEGG pathway analyses for each module separately to investigate the KEGG pathways affected by each module. We used the statistical method of hypergeometric test to select pathways with P- and Q-values <0.05, as significantly enriched pathways, and we found that the blue module predominantly affected pathways related to cell cycle and cell division. In the brown module, genes mainly affected a series of signaling pathways involved in the metabolism of multiple compounds. The green module mainly functions as a biological pathway that affects cell differentiation and phagocytosis. The turquoise module mainly functions in BPs affecting cell junctions and ECM-receptor interactions. Finally, the yellow module plays an important role in the complement and coagulation cascades of the organism, *Staphylococcus aureus* infection response, and cytokine and cytokine receptor interactions. Meanwhile, we performed GO and KEGG analyses on hub genes in the red module, which are shown in Supplementary Figure S1.

### ROC Analysis of the Selected Five Hub Genes

**FIGURE 7:**
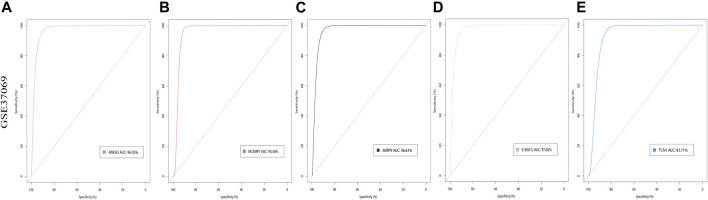
The ROC curve of five hub genes in GSE37069 and GSE139028. **(A–E)** The ROC curve of ANXA3, MCEMP1, MMP9, S100A12, and TCN1 in GSE37069. The x-axis shows specificity, and the y-axis shows sensitivity. ROC, receiver operating characteristic; AUC, area under the ROC curve.

Next, we selected the five most critical burn-related hub genes, *ANXA3*, *MCEMP1*, *MMP9*, *S100A12*, and *TCN1*, according to the principle that the expression differences in hub genes were significant and co-occurred in the PPI network and in the enrichment results. Based on the expression data of these genes in GSE37069 and GSE13902 separately, the ROC curve was plotted to assess the diagnostic value of the genes for burn injury (Figure 7). Figures 7A–E depicts the results of ROC validation analysis of the five hub genes *ANXA3* (AUC = 96.05%), *MCEMP1* (AUC = 95.56%), *MMP9* (AUC = 96.63%), *S100A12* (AUC = 97.06%), and *TCN1* (AUC = 93.11%) in the GSE37069 validation dataset.

### Validation of Expression Levels of 5 Hub Genes in Four Burn-Related Databases

**FIGURE 8:**
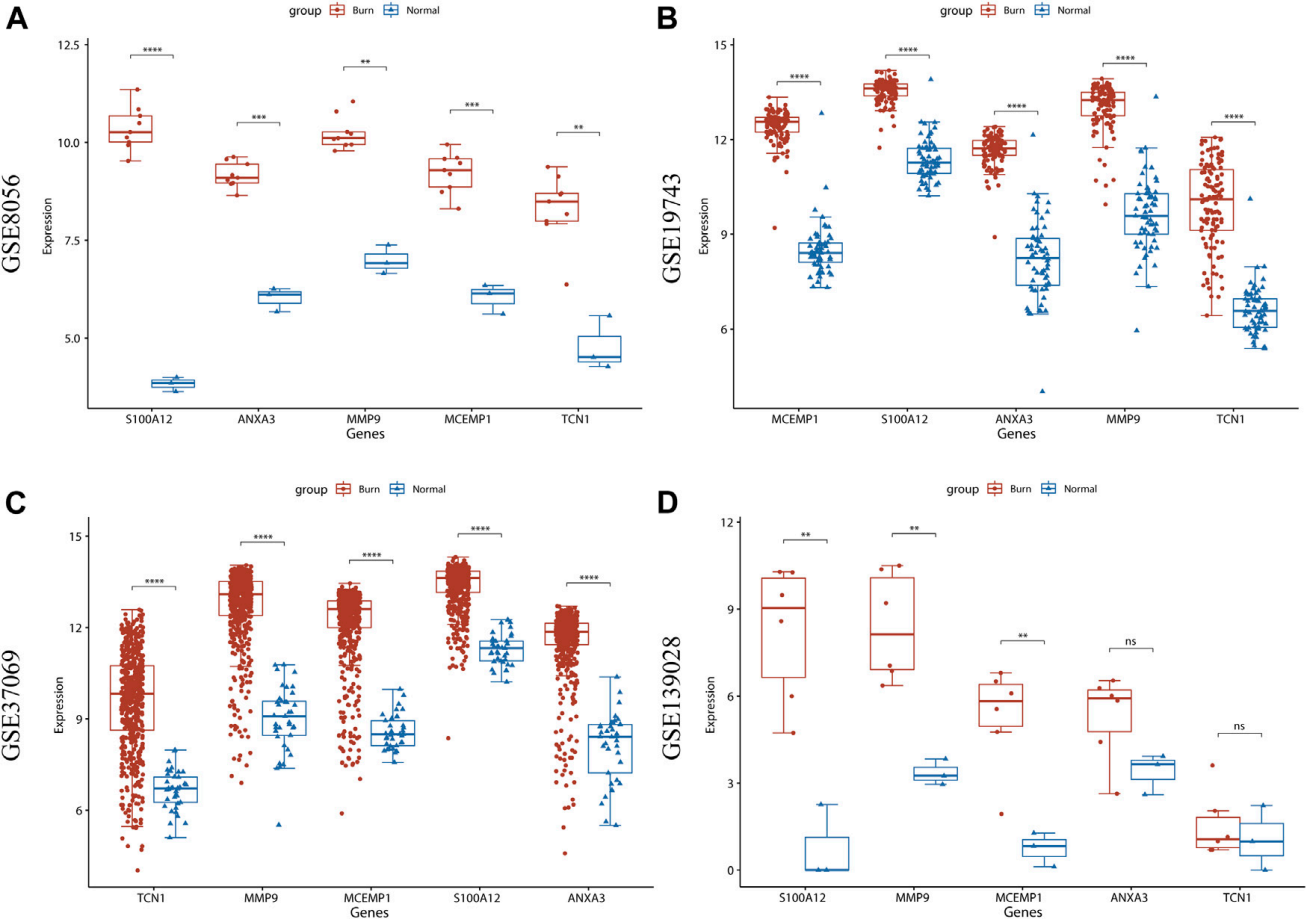
Validation of expression levels of 5 hub genes in 4 burn-related GEO datasets. **(A)** The expression level of ANXA3, MCEMP1, MMP9, S100A12, and TCN1 between burned patients and normal patients in GSE8056. **(B)** The expression level of ANXA3, MCEMP1, MMP9, S100A12, and TCN1 between burned patients and normal patients in GSE19743. **(C)** The expression level of ANXA3, MCEMP1, MMP9, S100A12, and TCN1 between burned patients and normal patients in GSE37069. **(D)** The expression level of ANXA3, MCEMP1, MMP9, S100A12, and TCN1 between burned patients and normal patients from GSE139028. The red boxplot indicates the burn patient group, and the blue boxplot indicates the normal sample group. A t-test was performed to compare the means of the two groups (* represents *p* < 0.05, ** represents *p* < 0.01, *** represents *p* < 0.001, **** represents *p* < 0.0001, ns represents not significant).

*ANXA3*, *MCEMP1*, *MMP9*, *S100A12*, and *TCN1* were identified as the key hub genes for burn status and were selected for subsequent analysis. We further visualized the expression of these genes in the GSE123568 dataset and found that the expression levels of these genes were significantly higher in the burn group than in the normal group (*p* < 0.05, Figure 8A). The expression patterns were also verified using GSE19743 (Figure 8B), GSE37069 (Figure 8C), and GSE139028 (Figure 8D) datasets, and the results revealed that the expression levels of the five hub genes were constantly increased in the burn tissues (*p* < 0.05).

### Preliminary IHC Validation

**FIGURE 9:**
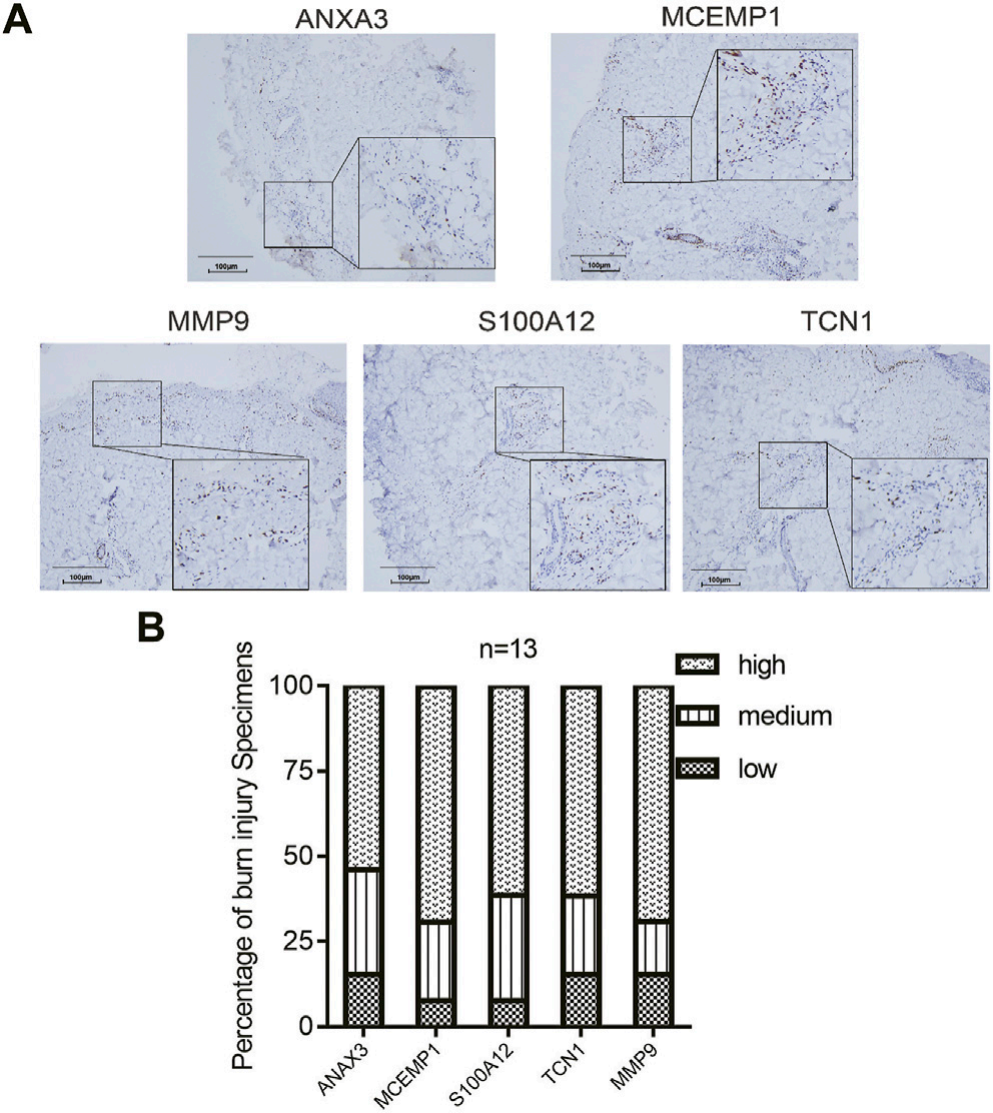
Expression of the hub genes in burn injury skin tissues. **(A)** Representative images of immunohistochemical expression of ANXA3, MCEMP1, MMP9, S100A12, and TCN1 in deep second-degree burn skin tissues from second-degree burn patients (*N* = 13). **(B)** The histogram demonstrated that ANXA3, MCEMP1, S100A12, TCN1, and MMP9 showed positive expression in second-degree burn samples.

IHC staining was performed. These five genes showed significantly high expression in the epidermal tissues of burn patients (Figure 9), demonstrating that their significant changes in the early stages of burn injury may mediate the drastic changes in the immune microenvironment induced by burn injury and induce inflammatory responses.

### Construction of Drug Activity, Transcription Factors, and miRNA Regulatory Network of the Five Hub Genes

**FIGURE 10:**
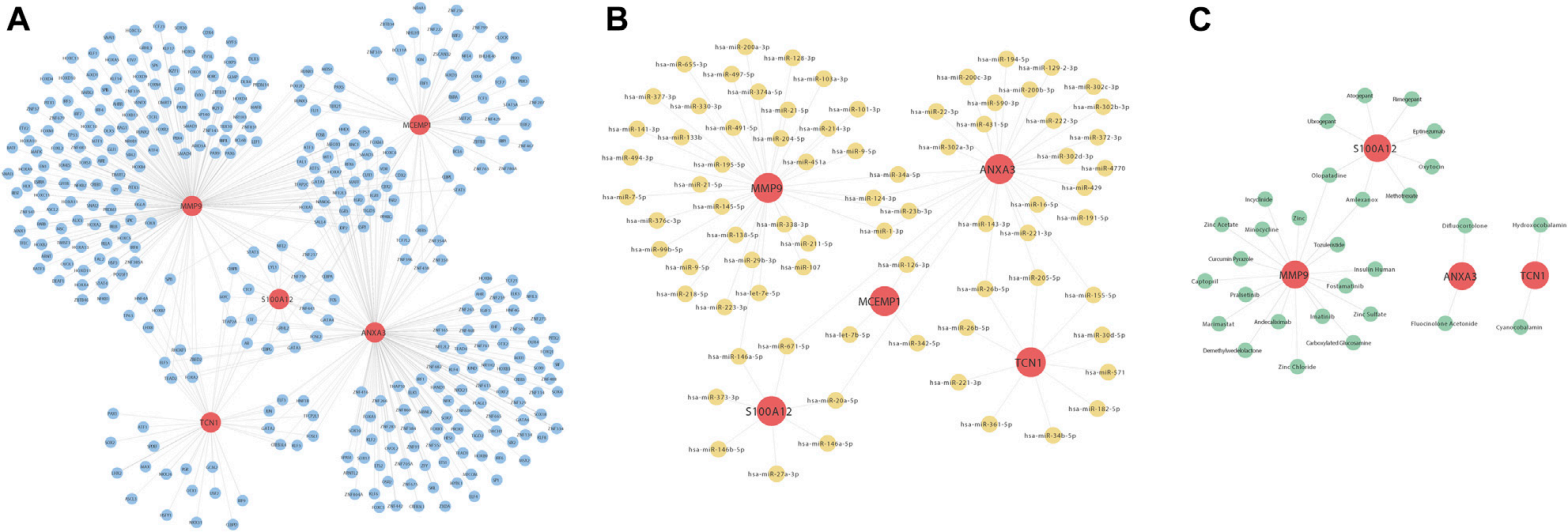
Construction of interaction network maps with transcription factors **(A)**, miRNAs **(B)**, and drug activity **(C)** for these five hub genes ANXA3, MCEMP1, MMP9, S100A12, and TCN1. **(A)** Interaction network diagram of the five hub genes and transcription factors, where red nodes represent key genes and blue nodes represent the transcription factors corresponding to the five hub genes. **(B)** Interaction network diagram of key genes and miRNAs, where red nodes represent key genes and yellow nodes represent miRNAs corresponding to the five hub genes. **(C)** Interaction network diagram of key genes and drug activities, where red nodes represent key genes and yellow nodes represent the drugs corresponding to the five hub genes.

We constructed the regulatory relationships of the target gene-drug interaction network using three drug-target databases, Drugbank, DGIdb, and PubChem. For target gene transcription factor analysis, we extracted and constructed a reciprocal relationship network between target genes and transcription factors through the ChEA3 website. Finally, we used three major miRNA and target gene databases, TarBase (V8.0), Starbase (V2.0), and miRTarbase, to extract the miRNA-target gene relationship based on the reciprocal relationship between miRNA and genes in the structure of miRNA-mRNA pairing relationships. The regulatory network of the above regulatory pairs was mapped and constructed using Cytoscape software (Figures 10A–C).

## Discussion

Burn injury is the leading cause of death around the world; however, the pathophysiology of burn wound tissue requires further investigation. Therefore, we aimed to explore the potential pathogenesis of burn-induced molecular changes in the peripheral blood of patients. We found that burn wound tissue-related genes with significant expression differences were mainly enriched in BPs such as immunity, epidermal development, and skin development, CCs such as collagen ECM, and MFs such as signal transduction receptor activity. KEGG pathway analysis showed significant enrichment of cytokine-cytokine receptor interaction, chemokine signaling pathway, ECM-receptor interaction, glutathione metabolism, osteoclast differentiation, and other pathways. To identify the modules that most strongly associated with genetic changes in burn wounds in blood samples from burned patients, we divided all genes into six separate modules through WGCNA algorithm analysis. The yellow gene module most closely associated with tissue damage in the burn process was also significantly positively correlated in the immune response of the organism, the emergency response to inflammation, and *epidermis* development. KEGG analysis showed an important role mainly in the complement and coagulation cascade of the organism, *S. aureus* infection, cytokine-cytokine receptor interactions, and other pathways.

Next, based on the principles of the 1. differential expression of hub genes, 2. co-occurrence in the PPI network, and 3. enrichment results, the five hub genes in the key yellow module were identified as the key genes for burn injury status, namely, *ANXA3*, *MCEMP1*, *MMP9*, *S100A12*, and *TCN1*. To verify the results of bioinformatics analysis, we performed ROC curve analysis to evaluate the diagnostic value of the five genes. The results demonstrated that these genes might serve as diagnostic markers for burn injury, because the AUC of these five genes was >0.9 in the GSE37069 validation dataset.

ANXA3, a member of the calcium-dependent phospholipid-binding protein family, has been shown to be involved in cellular growth and signal transduction pathways (Wang et al., 2019; Guo et al., 2021; Yang et al., 2021). Nevertheless, no previous study has documented the role of ANXA3 in burn injury. MCEMP1, a single-pass transmembrane protein, exerts profound influence in regulating mast cell differentiation and immune responses (Li et al., 2005). It was determined, for the first time, that the high expression of MCEMP1 in peripheral blood might serve as a prognostic biomarker of stroke (Raman et al., 2016) and that it plays a role in the pathogenesis of inflammation (Li et al., 2005) and sepsis (Xie et al., 2020). These previous studies demonstrated that MCEMP1 is a key regulator of several inflammation-related diseases. However, whether MCEMP1 is associated with burn injury remains unclear. MMP9 is involved in the degradation of the ECM in BPs, such as reproduction (Wang et al., 2021) and tissue remodeling (Nandi et al., 2020). Burn injuries can trigger tissue changes that can explain the variation in the levels of different biochemical markers that can be recorded both locally and systemically. Certain events observed in burn wounds, such as vascular hyperpermeability, have been associated with MMP released after trauma (Nagy et al., 2015). Furthermore, numerous studies have reported that the chronic inflammatory-induced secretion of MMP9 accelerates inner tissue remodeling after thermal burn injury (Stanciu et al., 2021), which is strongly correlated with the worsening result. It has been reported that S100A12, an epidermal pro-inflammatory cytokine, induced the formation of a hypertrophic scar (Zhao et al., 2017). Therefore, we hypothesized that S100A12 may be involved in the occurrence of burns because it is involved in the formation of skin scarring after burns. TCN1, a member of the vitamin B12-binding protein family, is responsible for the formation of secondary granules in neutrophils and facilitates the transport of cobalamin into cells. It was demonstrated by microarray and experimental methods that TCN1 and S100A12 may affect the disease process of acne by participating in the innate immune and cellular differentiation processes of hair follicles and epidermal keratin-forming cells (Zouboulis et al., 2020). These studies suggest that TCN1 and S100A12 may participate in burn injury. Notably, the expression of these five hub genes was verified in the GSE37069 and GSE139028 datasets, and this expression was significantly increased in the burn group in all four GEO datasets. In addition to developing and validating these five key pivotal genes associated with burn diagnosis, we have also predicted target drugs, transcription factors, and regulatory microRNAs associated with these five burn diagnostic markers, and these predicted target drugs and molecules provide new targets for burn treatment. We should conduct future molecular experiments to identify their role in burn treatment.

In conclusion, using multiple data set analysis, we identified genetic changes in epidermal tissue after burns and the biological functions of this process, and screened the module most relevant to skin damage based on WGCNA analysis in the blood sample dataset of burn patients and analyzed its MFs. Then, we further screened five key genes in the module within that module and then performed expression and ROC analysis to validate their diagnostic efficacy. Finally, preliminary IHC validation of the five hub genes was performed in the epidermal tissues of burn patients.

## Conclusion

There were several highlights to the current study. First, few studies have focused on identifying diagnostic biomarkers in peripheral blood for burn skin injuries. Skin tissue expression profiles of healthy individuals and burn patients help us to gain a comprehensive understanding of their pathological processes and to identify diagnostic biomarkers for burn injuries. Second, WGCNA has a particular advantage in processing gene expression datasets because it can estimate the connectivity between modules and clinical features. However, this study had some limitations. Here, we validated the expression level and diagnostic efficacy of five potential diagnostic biomarkers identified on a dataset of blood samples from real burn patients and on skin tissue. However, the number of clinical cases we validated was small. The specific roles of these biomarkers in burns, their specific associations with clinical features, such as burn extent and scar healing, and their diagnostic roles require further investigation and validation in a larger number of clinical patients.

## Data Availability

The original contributions presented in the study are included in the article/Supplementary Material. Further inquiries can be directed to the corresponding authors.

## Supplementary Material

Supplement Figure S1 The KEGG and GO enrichment of the hub genes. **(A)** Chord diagram showed the results of enrichment analysis of hub genes, each legend represents a different GO pathway (BP, CC or MF), and the color of the left outer circle represents the upregulated (red) and downregulated (blue) genes. **(B)** Bar diagram showed the enrichment analysis of KEGG pathway of the genes.

## Abbreviations

WGCNA, weighted gene co-expression network analysis; PPI, protein-protein interaction; FC, fold change; ROC, receiver operating characteristic; DEGs, differentially expressed genes; GO, gene ontology; GSEA, gene set enrichment analysis; KEGG, Kyoto Encyclopedia of Genes and Genomes; AUC, area under curve; NES, normalized enrichment score.

## References

[B1] Auger C. Samadi O. Jeschke M. G. (2017). The Biochemical Alterations Underlying post-burn Hypermetabolism. Biochim. Biophys. Acta Mol. Basis Dis. 1863 (10 Pt B), 2633–2644. 10.1016/j.bbadis.2017.02.019 28219767 PMC5563481

[B2] Barrett T. Wilhite S. E. Ledoux P. Evangelista C. Kim I. F. Tomashevsky M. (2013). NCBI GEO: Archive for Functional Genomics Data Sets-Update. Nucleic Acids Res. 41, D991–D995. 10.1093/nar/gks1193 23193258 PMC3531084

[B3] Cotto K. C. Wagner A. H. Feng Y.-Y. Kiwala S. Coffman A. C. Spies G. (2018). DGIdb 3.0: a Redesign and Expansion of the Drug-Gene Interaction Database. Nucleic Acids Res. 46 (D1), D1068–d1073. 10.1093/nar/gkx1143 29156001 PMC5888642

[B4] Greenhalgh D. G. (2019). Management of Burns. N. Engl. J. Med. 380 (24), 2349–2359. 10.1056/NEJMra1807442 31189038

[B5] Guo C. Li N. Dong C. Wang L. Li Z. Liu Q. (2021). 33-kDa ANXA3 Isoform Contributes to Hepatocarcinogenesis via Modulating ERK, PI3K/Akt-HIF and Intrinsic Apoptosis Pathways. J. Adv. Res. 30, 85–102. 10.1016/j.jare.2020.11.003 34026289 PMC8132212

[B6] Haagsma J. A. Graetz N. Bolliger I. Naghavi M. Higashi H. Mullany E. C. (2016). The Global burden of Injury: Incidence, Mortality, Disability-Adjusted Life Years and Time Trends from the Global Burden of Disease Study 2013. Inj. Prev. 22 (1), 3–18. 10.1136/injuryprev-2015-041616 26635210 PMC4752630

[B7] Hu Y. Yan C. Hsu C.-H. Chen Q.-R. Niu K. Komatsoulis G. A. (2014). OmicCircos: A Simple-To-Use R Package for the Circular Visualization of Multidimensional Omics Data. Cancer Inform. 13, CIN.S13495–20. 10.4137/cin.S13495 PMC3921174 24526832

[B8] Huang H.-Y. Lin Y.-C. -D. Li J. Huang K.-Y. Shrestha S. Hong H.-C. (2020). miRTarBase 2020: Updates to the Experimentally Validated microRNA-Target Interaction Database. Nucleic Acids Res. 48 (D1), D148–d154. 10.1093/nar/gkz896 31647101 PMC7145596

[B9] Karagkouni D. Paraskevopoulou M. D. Chatzopoulos S. Vlachos I. S. Tastsoglou S. Kanellos I. (2018). DIANA-TarBase V8: a Decade-Long Collection of Experimentally Supported miRNA-Gene Interactions. Nucleic Acids Res. 46 (D1), D239–d245. 10.1093/nar/gkx1141 29156006 PMC5753203

[B10] Keenan A. B. Torre D. Lachmann A. Leong A. K. Wojciechowicz M. L. Utti V. (2019). ChEA3: Transcription Factor Enrichment Analysis by Orthogonal Omics Integration. Nucleic Acids Res. 47 (W1), W212–w224. 10.1093/nar/gkz446 31114921 PMC6602523

[B11] Langfelder P. Horvath S. (2008). WGCNA: an R Package for Weighted Correlation Network Analysis. BMC Bioinformatics 9, 559. 10.1186/1471-2105-9-559 19114008 PMC2631488

[B12] Lee S. RahulYe H. Ye H. Chittajallu D. Kruger U. Boyko T. (2020). Real-time Burn Classification Using Ultrasound Imaging. Sci. Rep. 10 (1), 5829. 10.1038/s41598-020-62674-9 32242131 PMC7118155

[B13] Li J.-H. Liu S. Zhou H. Qu L.-H. Yang J.-H. (2014). starBase v2.0: Decoding miRNA-ceRNA, miRNA-ncRNA and Protein-RNA Interaction Networks from Large-Scale CLIP-Seq Data. Nucl. Acids Res. 42, D92–D97. 10.1093/nar/gkt1248 24297251 PMC3964941

[B14] Li K. Wang S.-W. Li Y. Martin R. E. Li L. Lu M. (2005). Identification and Expression of a New Type II Transmembrane Protein in Human Mast Cells. Genomics 86 (1), 68–75. 10.1016/j.ygeno.2005.03.006 15953541

[B15] McGill D. J. Sørensen K. MacKay I. R. Taggart I. Watson S. B. (2007). Assessment of Burn Depth: a Prospective, Blinded Comparison of Laser Doppler Imaging and Videomicroscopy. Burns 33 (7), 833–842. 10.1016/j.burns.2006.10.404 17614206

[B16] Nagy B. Szélig L. Rendeki S. Loibl C. Rézmán B. Lantos J. (2015). Dynamic Changes of Matrix Metalloproteinase 9 and Tissue Inhibitor of Metalloproteinase 1 after Burn Injury. J. Crit. Care 30 (1), 162–166. 10.1016/j.jcrc.2014.07.008 25155253

[B17] Nandi S. S. Katsurada K. Sharma N. M. Anderson D. R. Mahata S. K. Patel K. P. (2020). MMP9 Inhibition Increases Autophagic Flux in Chronic Heart Failure. Am. J. Physiol. Heart Circulatory Physiol. 319 (6), H1414–h1437. 10.1152/ajpheart.00032.2020 PMC7792705 33064567

[B18] Niggemann P. Rittirsch D. Buehler P. K. Schweizer R. Giovanoli P. Reding T. (2021). Incidence and Time Point of Sepsis Detection as Related to Different Sepsis Definitions in Severely Burned Patients and Their Accompanying Time Course of Pro-inflammatory Biomarkers. JPM 11 (8), 701. 10.3390/jpm11080701 34442346 PMC8401386

[B19] Ou S. Liu G.-D. Tan Y. Zhou L.-S. Bai S.-R. Xue G. (2015). A Time Course Study about Gene Expression of post-thermal Injury with DNA Microarray. Int. J. Dermatol. 54 (7), 757–764. 10.1111/ijd.12534 25069606

[B20] Peterson J. R. De La Rosa S. Eboda O. Cilwa K. E. Agarwal S. Buchman S. R. (2014). Treatment of Heterotopic Ossification through Remote ATP Hydrolysis. Sci. Transl. Med. 6 (255), 255ra132. 10.1126/scitranslmed.3008810 PMC4353731 25253675

[B21] Raman K. O’Donnell M. J. Czlonkowska A. Duarte Y. C. Lopez-Jaramillo P. Peñaherrera E. (2016). Peripheral Blood MCEMP1 Gene Expression as a Biomarker for Stroke Prognosis. Stroke 47 (3), 652–658. 10.1161/strokeaha.115.011854 26846866

[B22] Ritchie M. E. Phipson B. Wu D. Hu Y. Law C. W. Shi W. (2015). Limma powers Differential Expression Analyses for RNA-Sequencing and Microarray Studies. Nucleic Acids Res. 43 (7), e47. 10.1093/nar/gkv007 25605792 PMC4402510

[B23] Robin X. Turck N. Hainard A. Tiberti N. Lisacek F. Sanchez J.-C. (2011). pROC: an Open-Source Package for R and S+ to Analyze and Compare ROC Curves. BMC Bioinformatics 12, 77. 10.1186/1471-2105-12-77 21414208 PMC3068975

[B24] Schutte S. C. Evdokiou A. Satish L. (2020). Protease Levels Are Significantly Altered in Pediatric Burn Wounds. Burns 46, 1603–1611. 10.1016/j.burns.2020.04.037 32482377

[B25] Shanahan H. P. Memon F. N. Upton G. J. G. Harrison A. P. (2012). Normalized Affymetrix Expression Data Are Biased by G-Quadruplex Formation. Nucleic Acids Res. 40 (8), 3307–3315. 10.1093/nar/gkr1230 22199258 PMC3333884

[B26] Shannon P. Markiel A. Ozier O. Baliga N. S. Wang J. T. Ramage D. (2003). Cytoscape: a Software Environment for Integrated Models of Biomolecular Interaction Networks. Genome Res. 13 (11), 2498–2504. 10.1101/gr.1239303 14597658 PMC403769

[B27] Stanciu A. Zamfir-Chiru-Anton A. Stanciu M. Gherghe M. Hainarosie R. Furtunescu F. (2021). Role and Dynamics of Matrix Metalloproteinase 9 and Tissue Inhibitor of Metalloproteinase 1 in Burn Patients. Exp. Ther. Med. 22 (4), 1062. 10.3892/etm.2021.10496 34434276 PMC8353633

[B28] Wang J. Jia X. Meng X. Li Y. Wu W. Zhang X. (2019). Annexin A3 May Play an Important Role in Ochratoxin-Induced Malignant Transformation of Human Gastric Epithelium Cells. Toxicol. Lett. 313, 150–158. 10.1016/j.toxlet.2019.07.002 31276768

[B29] Wang S. Shi C. Cai X. Wang Y. Chen X. Han H. (2021). Human Acellular Amniotic Matrix with Previously Seeded Umbilical Cord Mesenchymal Stem Cells Restores Endometrial Function in a Rat Model of Injury. Mediators Inflamm. 2021, 1–14. 10.1155/2021/5573594 PMC8438588 34531703

[B30] Wang Y. Bryant S. H. Cheng T. Wang J. Gindulyte A. Shoemaker B. A. (2017). PubChem BioAssay: 2017 Update. Nucleic Acids Res. 45 (D1), D955–d963. 10.1093/nar/gkw1118 27899599 PMC5210581

[B31] Wishart D. S. Feunang Y. D. Guo A. C. Lo E. J. Marcu A. Grant J. R. (2018). DrugBank 5.0: a Major Update to the DrugBank Database for 2018. Nucleic Acids Res. 46 (D1), D1074–d1082. 10.1093/nar/gkx1037 29126136 PMC5753335

[B32] Xie W. Chen L. Chen L. Kou Q. (2020). Silencing of Long Non-coding RNA MALAT1 Suppresses Inflammation in Septic Mice: Role of microRNA-23a in the Down-Regulation of MCEMP1 Expression. Inflamm. Res. 69 (2), 179–190. 10.1007/s00011-019-01306-z 31893303

[B33] Yang L. Lu P. Yang X. Li K. Qu S. (2021). Annexin A3, a Calcium-dependent Phospholipid-Binding Protein: Implication in Cancer. Front. Mol. Biosci. 8, 716415. 10.3389/fmolb.2021.716415 34355022 PMC8329414

[B34] Yu G. Wang L.-G. Han Y. He Q.-Y. (2012). clusterProfiler: an R Package for Comparing Biological Themes Among Gene Clusters. OMICS: A J. Integr. Biol. 16 (5), 284–287. 10.1089/omi.2011.0118 PMC3339379 22455463

[B35] Zhao J. Zhong A. Friedrich E. E. Jia S. Xie P. Galiano R. D. (2017). S100A12 Induced in the Epidermis by Reduced Hydration Activates Dermal Fibroblasts and Causes Dermal Fibrosis. J. Invest. Dermatol. 137 (3), 650–659. 10.1016/j.jid.2016.10.040 27840235

[B36] Zhou B. Xu W. Herndon D. Tompkins R. Davis R. Xiao W. (2010). Analysis of Factorial Time-Course Microarrays with Application to a Clinical Study of Burn Injury. Proc. Natl. Acad. Sci. U S A. 107 (22), 9923–9928. 10.1073/pnas.1002757107 20479259 PMC2890487

[B37] Zhou S. Ouyang W. Zhang X. Liao L. Pi X. Yang R. (2021). UTRN Inhibits Melanoma Growth by Suppressing P38 and JNK/c-Jun Signaling Pathways. Cancer Cel Int. 21 (1), 88. 10.1186/s12935-021-01768-4 PMC7905598 33632212

[B38] Zhu T. Su Q. Wang C. Shen L. Chen H. Feng S. (2021). SDF4 Is a Prognostic Factor for 28-Days Mortality in Patients with Sepsis via Negatively Regulating ER Stress. Front. Immunol. 12, 659193. 10.3389/fimmu.2021.659193 34326834 PMC8313857

[B39] Zouboulis C. C. Nogueira da Costa A. Makrantonaki E. Hou X. X. Almansouri D. Dudley J. T. (2020). Alterations in Innate Immunity and Epithelial Cell Differentiation Are the Molecular Pillars of Hidradenitis Suppurativa. J. Eur. Acad. Dermatol. Venereol. 34 (4), 846–861. 10.1111/jdv.16147 31838778

