# Supplementary figures and images for: Identification and Verification of Five Potential Biomarkers Related to Skin and Thermal Injury Using Weighted Gene Co-Expression Network Analysis
Source: Front Genet. 2022 Jan 3;12:781589. doi: 10.3389/fgene.2021.781589 (PMC8762241; doi:10.3389/fgene.2021.781589)

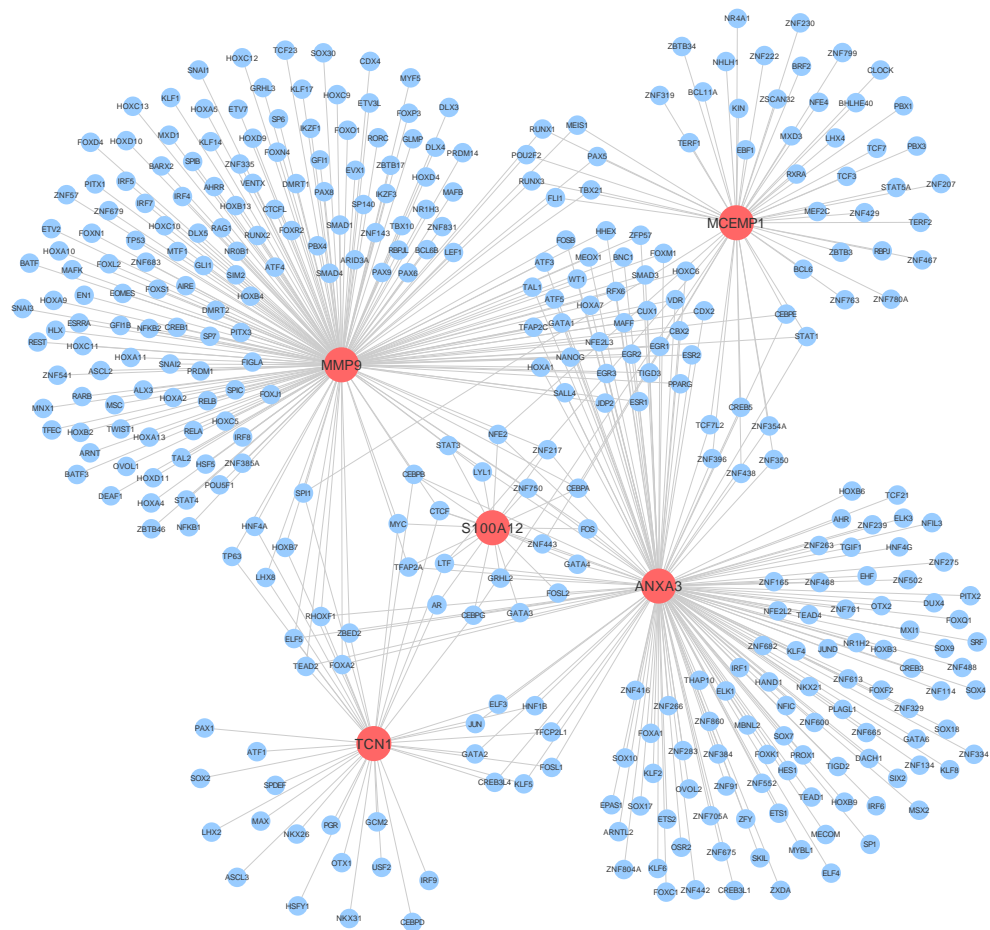

Supplement: Supplementary file 2 [file DataSheet11.ZIP › 11_Five_Gene_TF_Network_Analysis/Gene_TF_network.pdf]

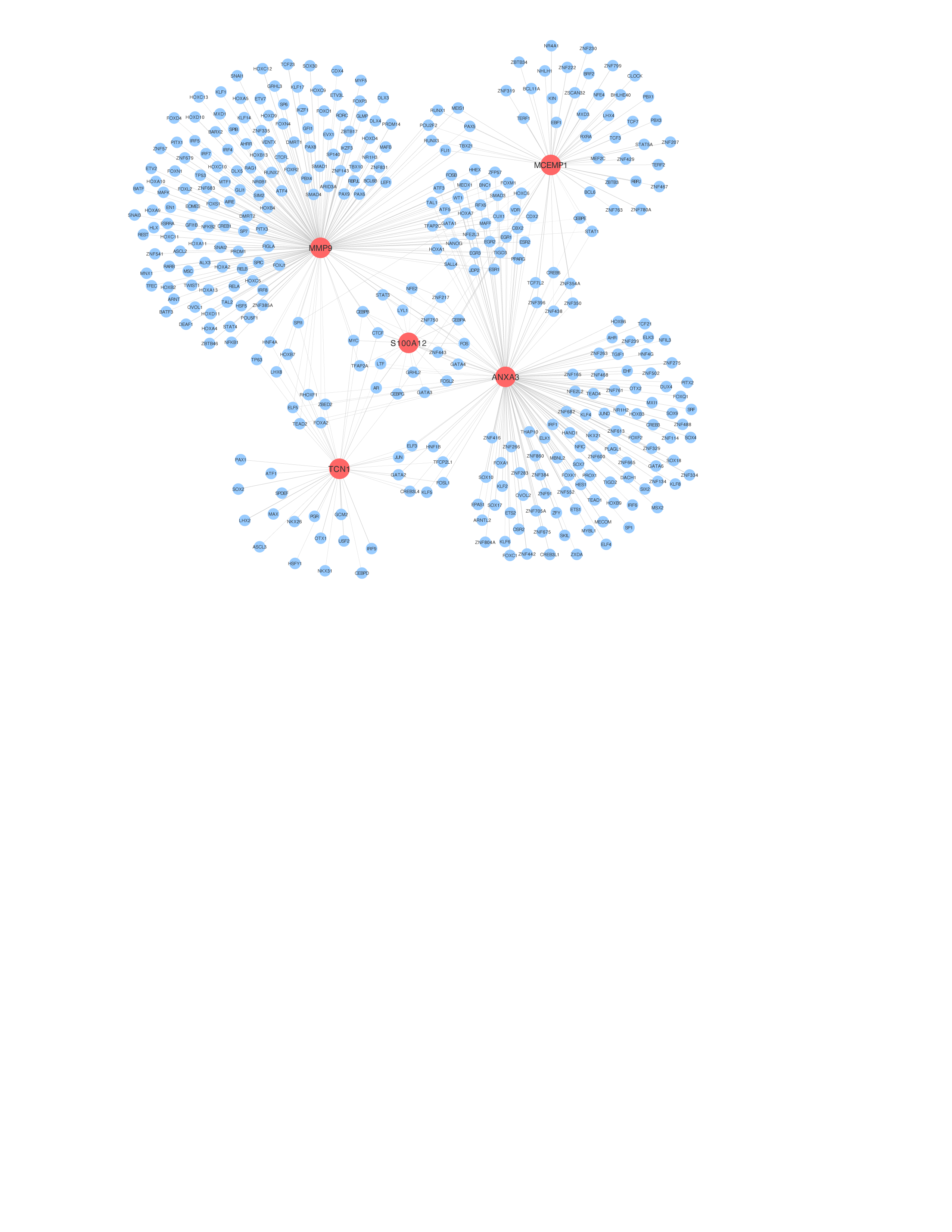

Supplement: Supplementary file 2 [file DataSheet11.ZIP › 11_Five_Gene_TF_Network_Analysis/Gene_TF_network.tif]

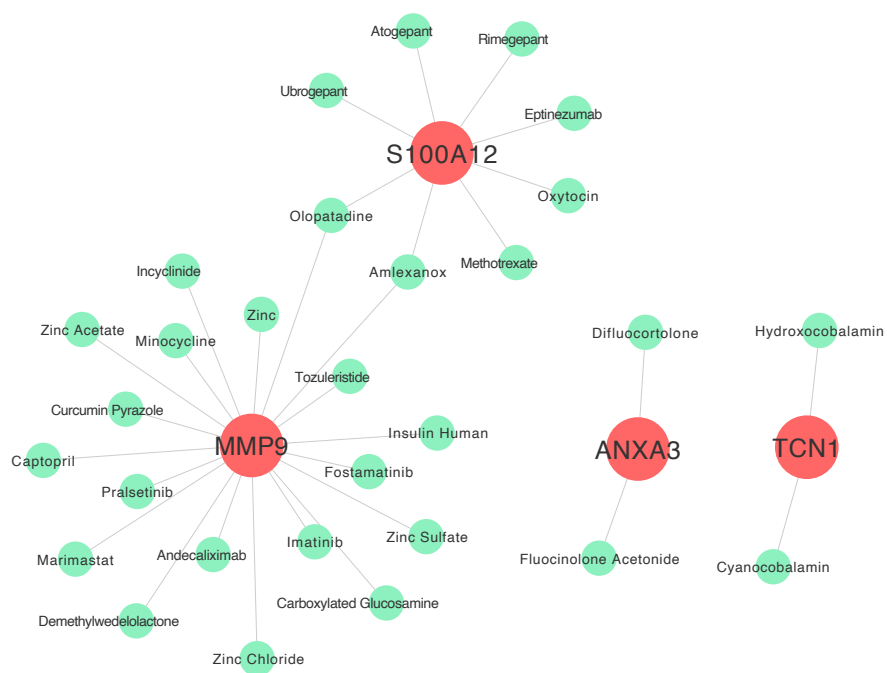

Supplement: Supplementary file 4 [file DataSheet9.ZIP › 09_Five_Gene_Drug_Network_Analysis/Gene_Drug_network.pdf]

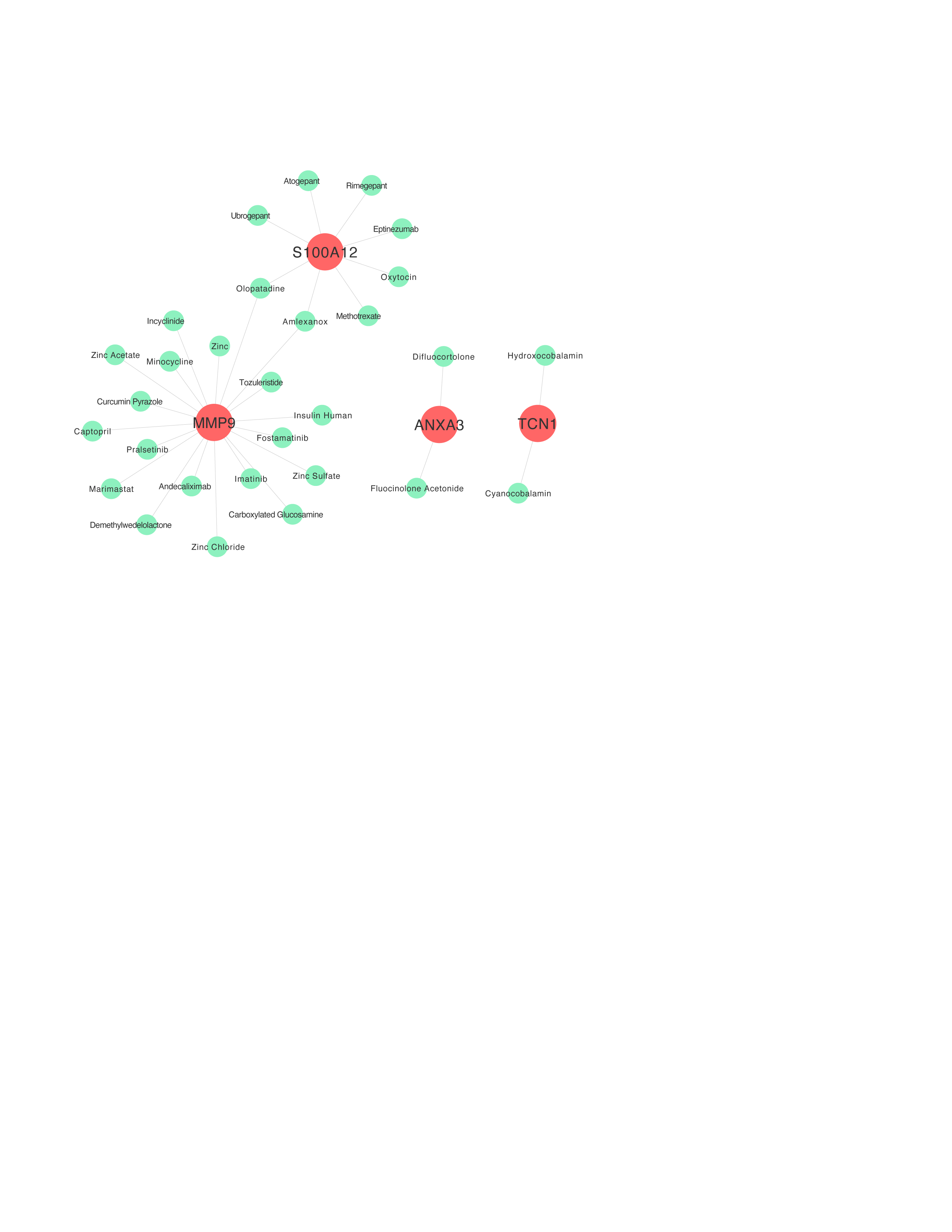

Supplement: Supplementary file 4 [file DataSheet9.ZIP › 09_Five_Gene_Drug_Network_Analysis/Gene_Drug_network.tif]

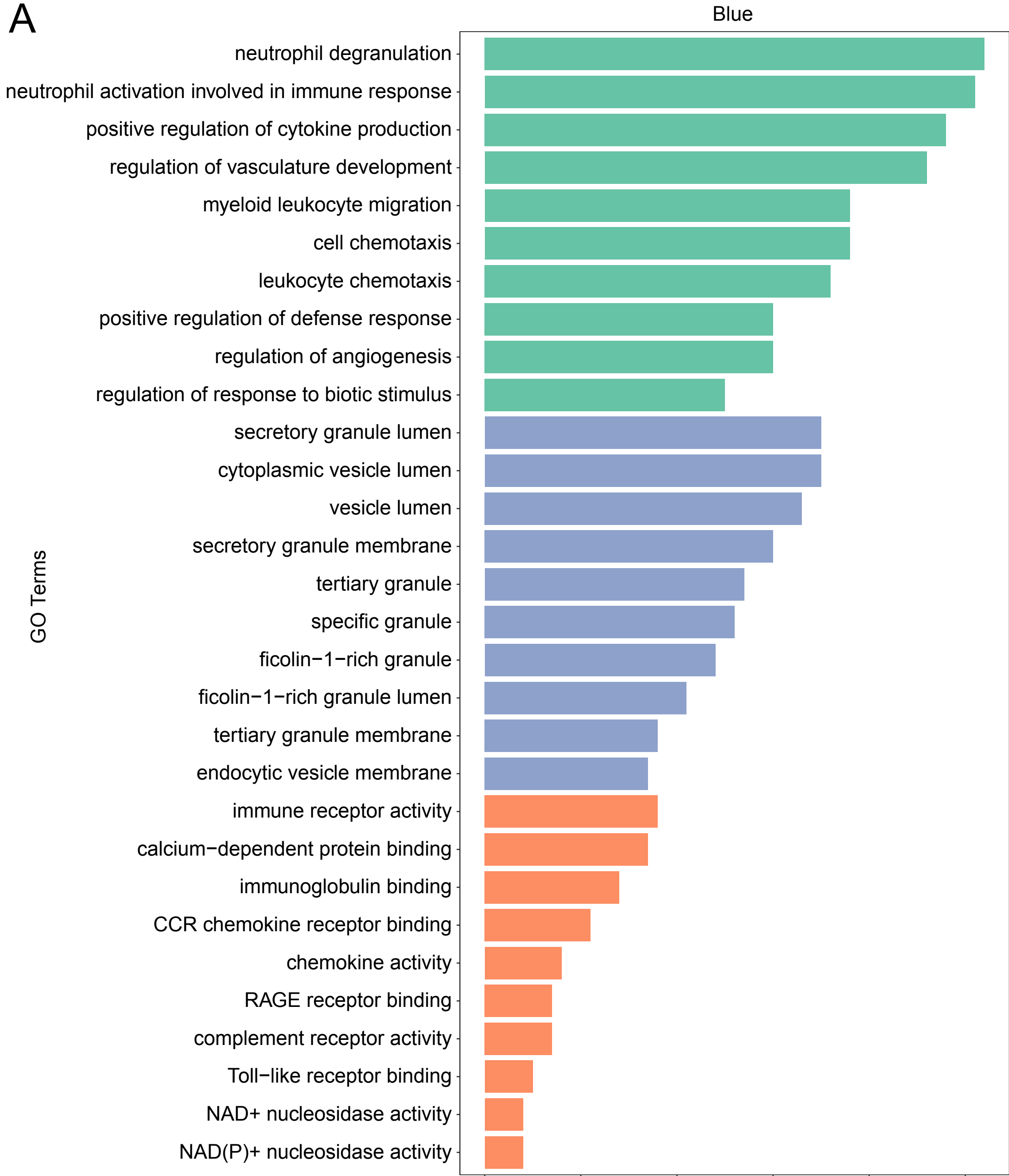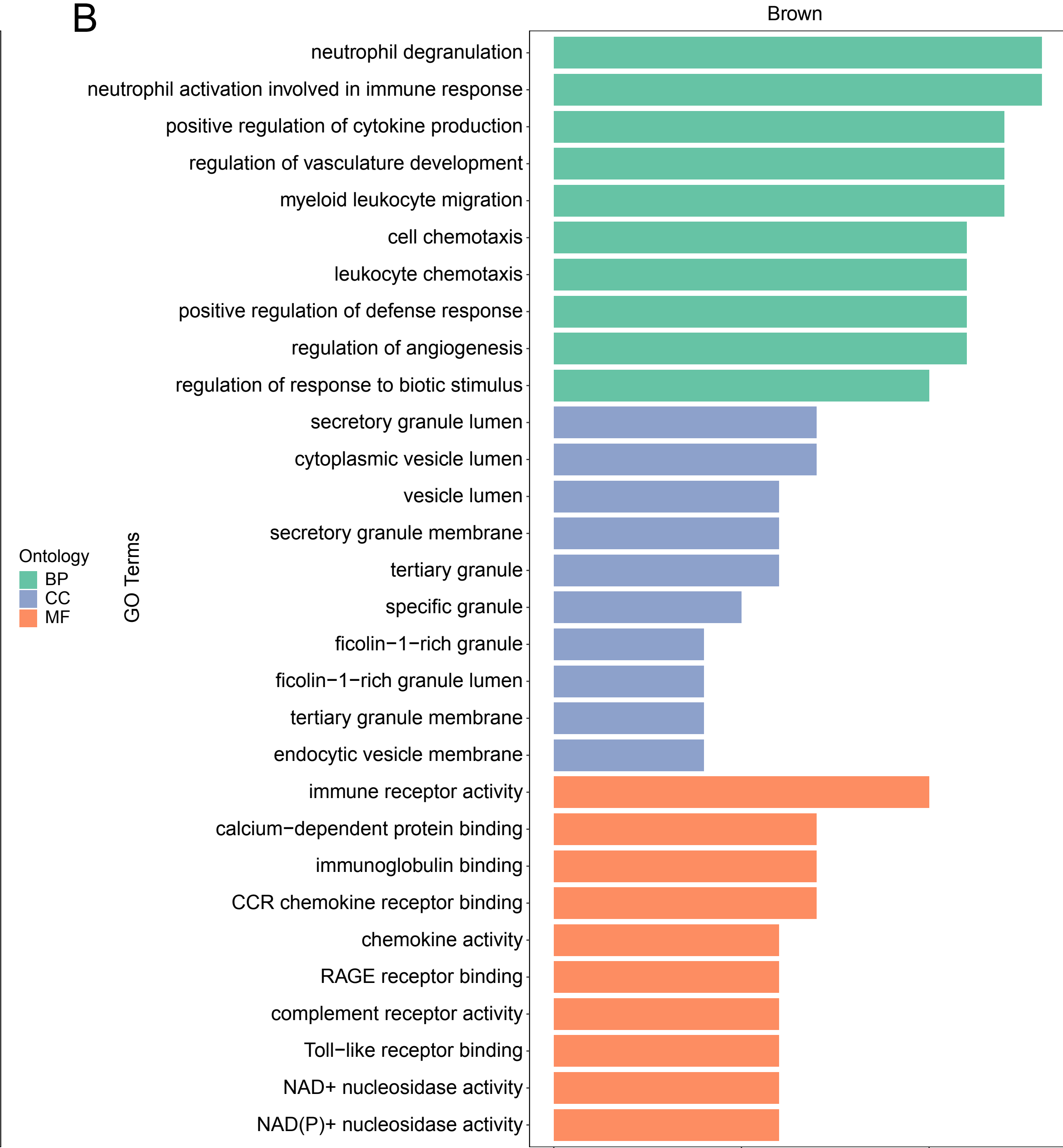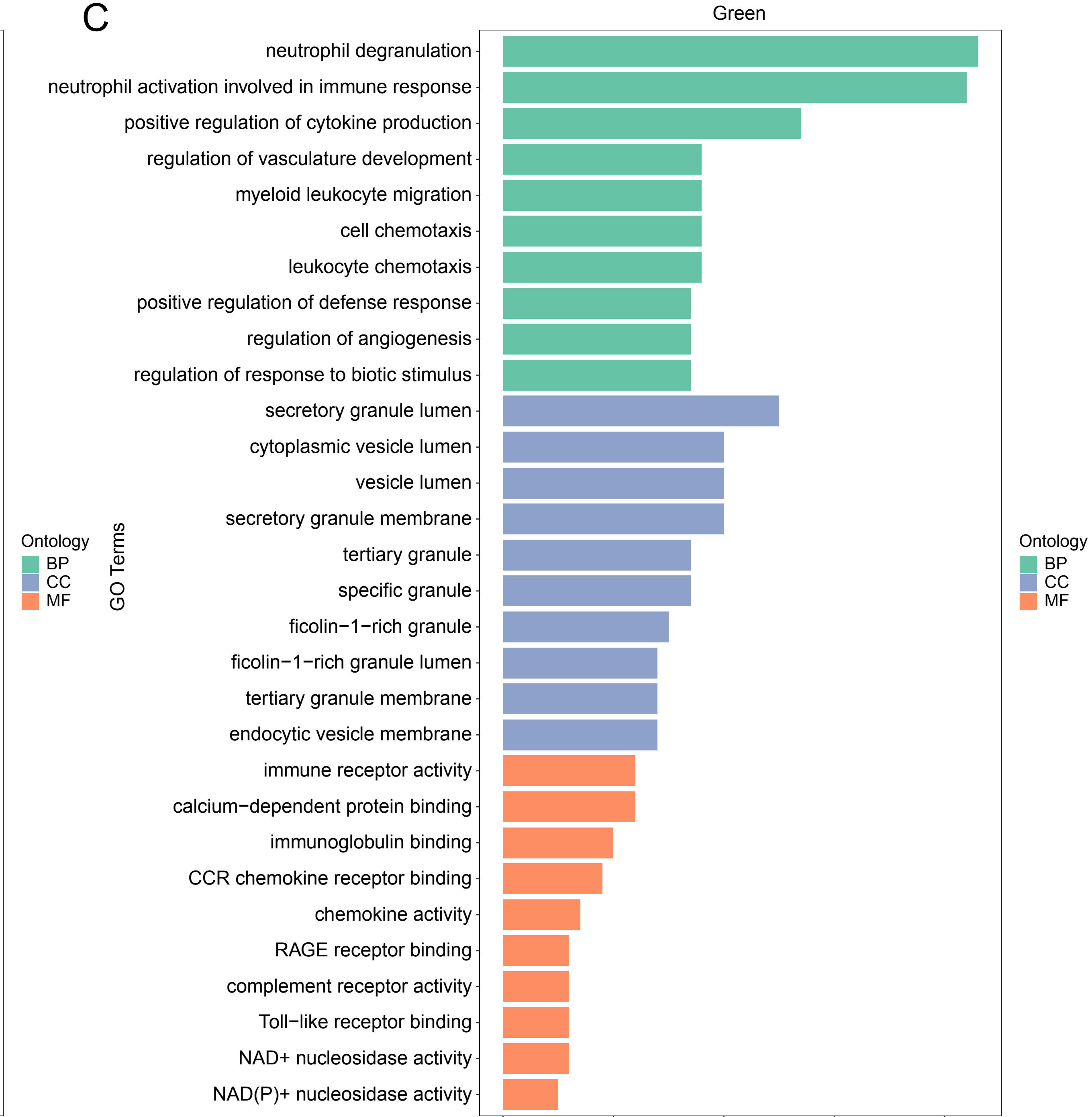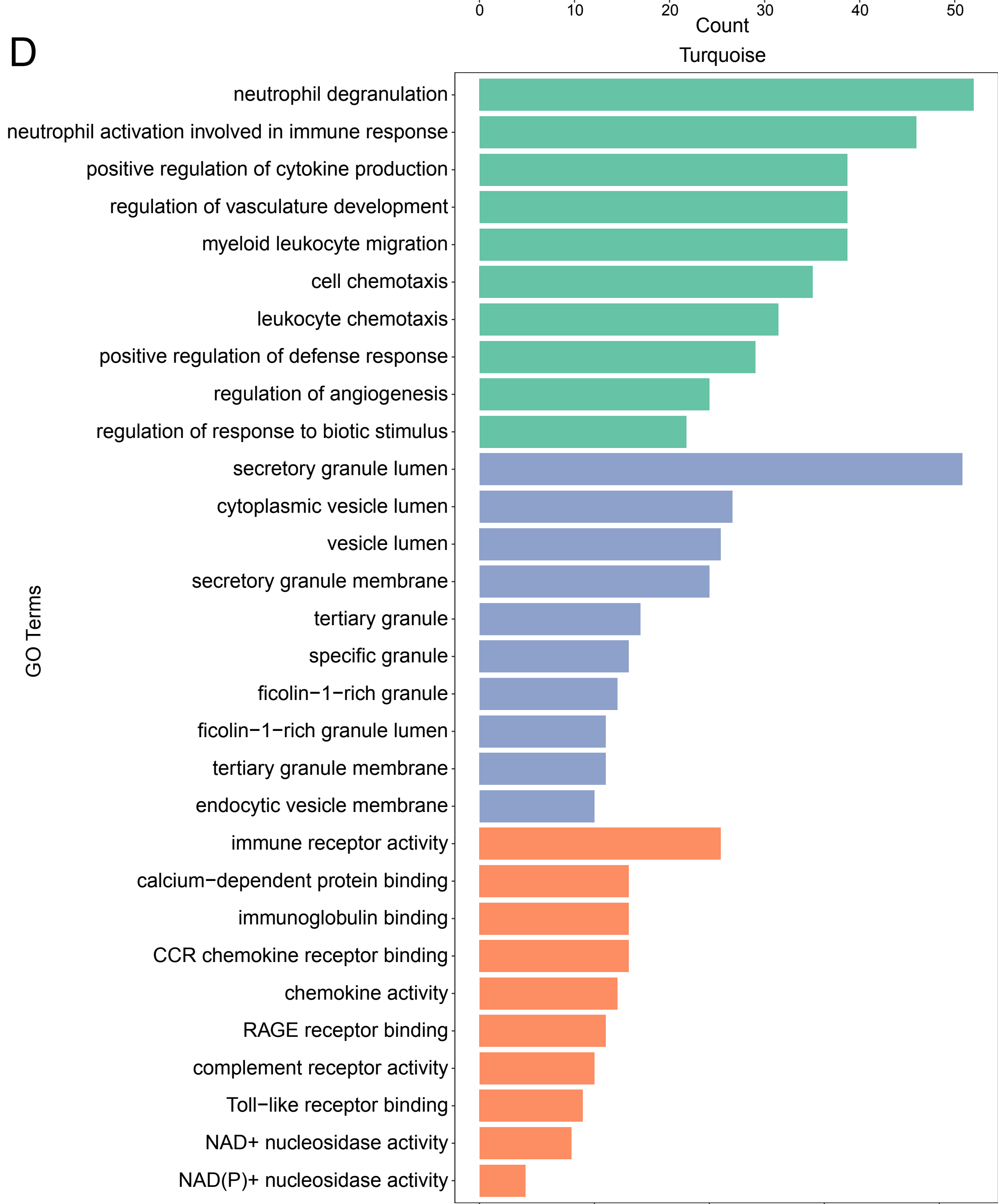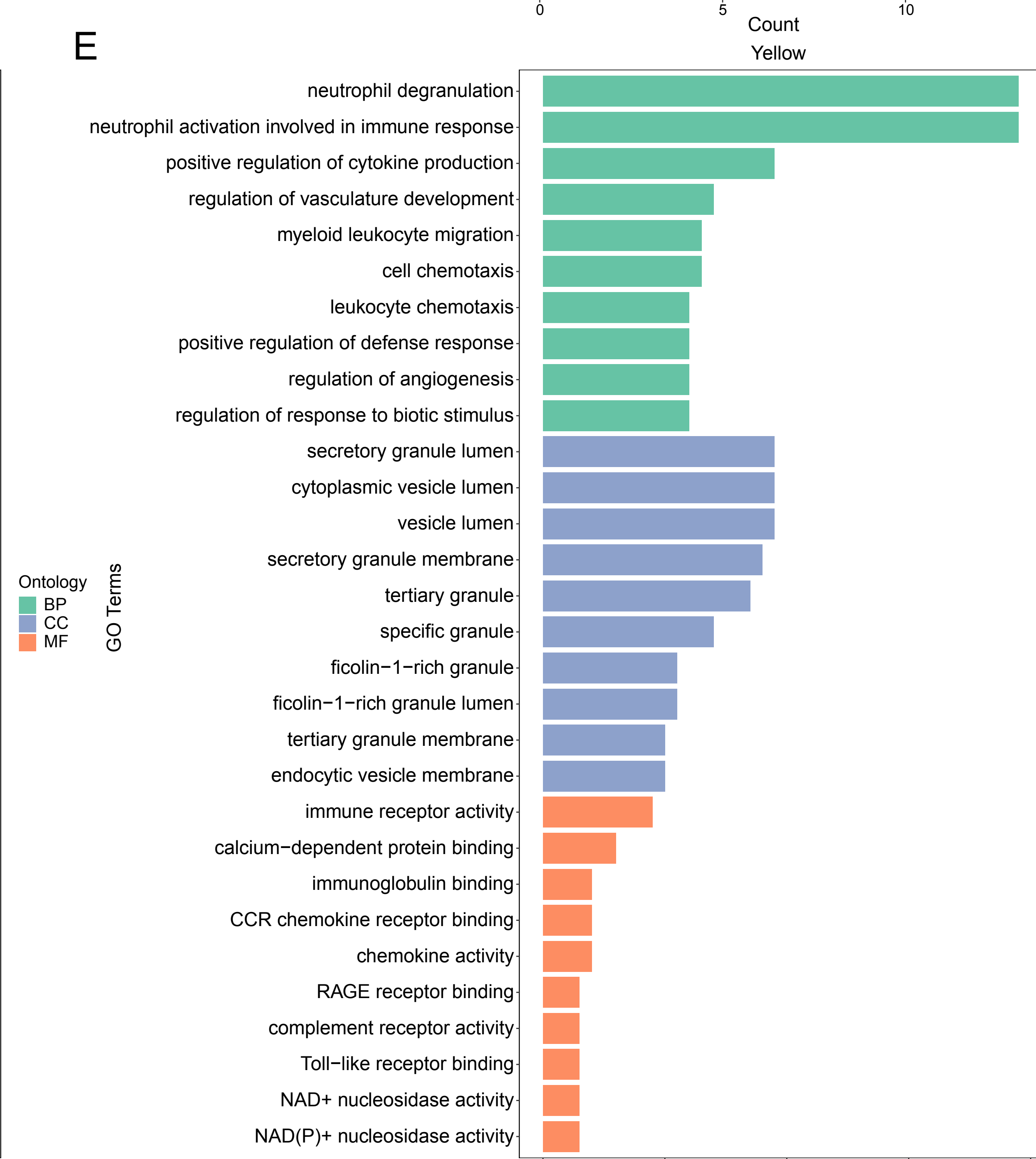

Supplement: Supplementary file 5 [file DataSheet4.ZIP › 04_Module_Gene_GO_KEGG/GO/Figure_5.pdf]

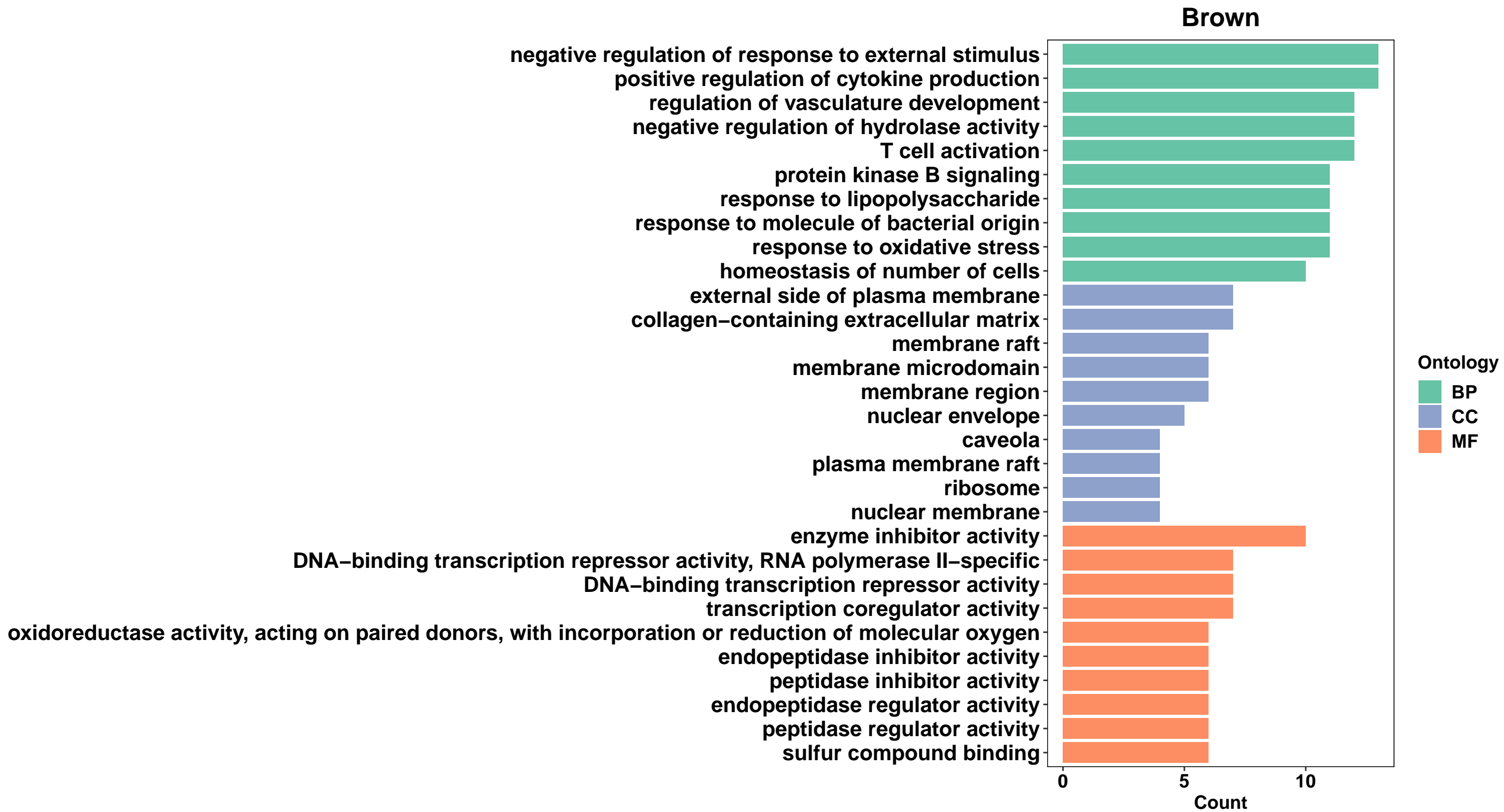

Supplement: Supplementary file 5 [file DataSheet4.ZIP › 04_Module_Gene_GO_KEGG/GO/brown_GO.pdf]

# Turquoise

GO Terms

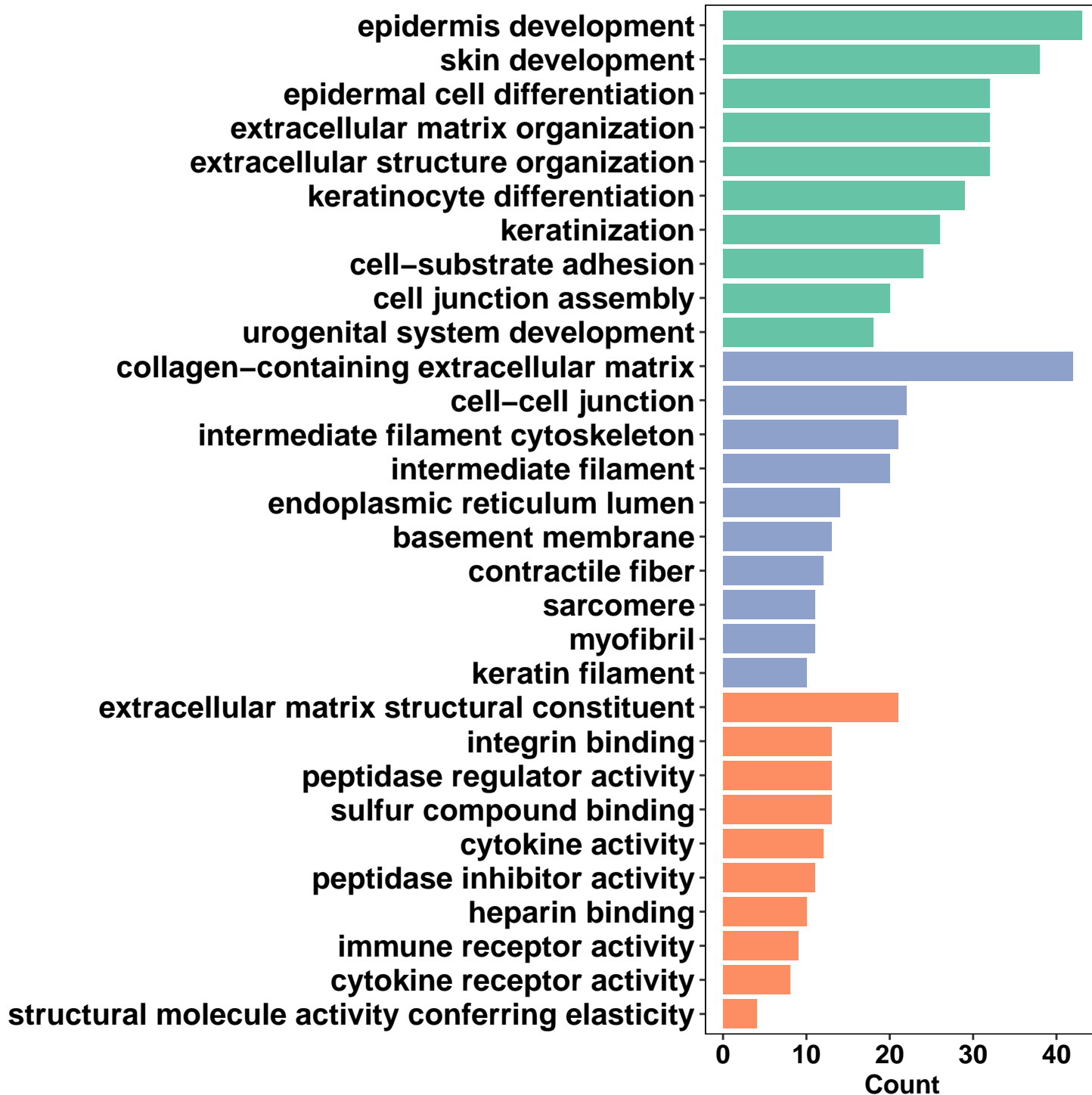

Ontology

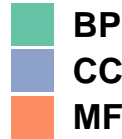

Supplement: Supplementary file 5 [file DataSheet4.ZIP › 04_Module_Gene_GO_KEGG/GO/turquoise_GO.pdf]

Yellow

GO Terms

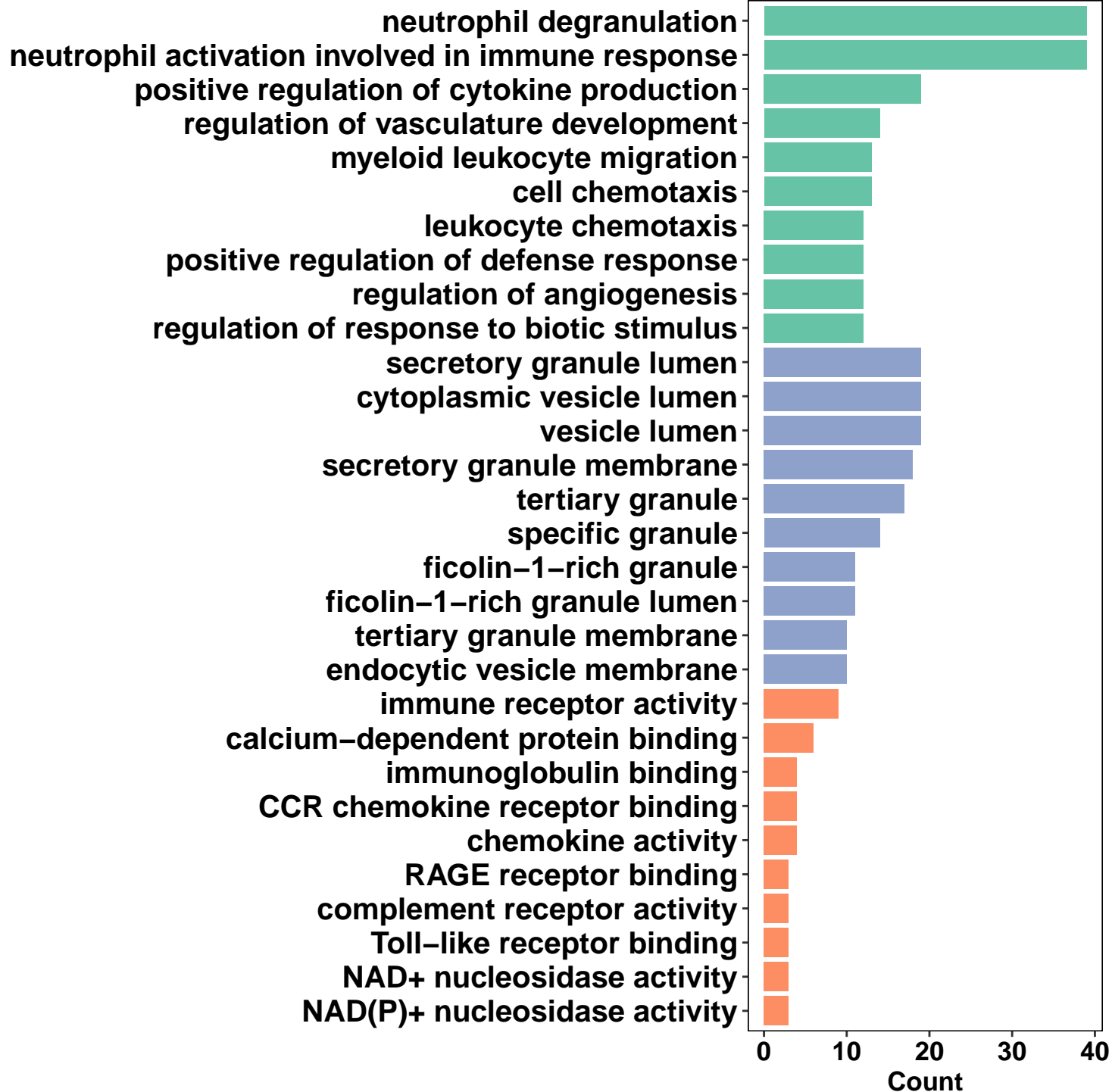

Supplement: Supplementary file 5 [file DataSheet4.ZIP › 04_Module_Gene_GO_KEGG/GO/yellow_GO.pdf]

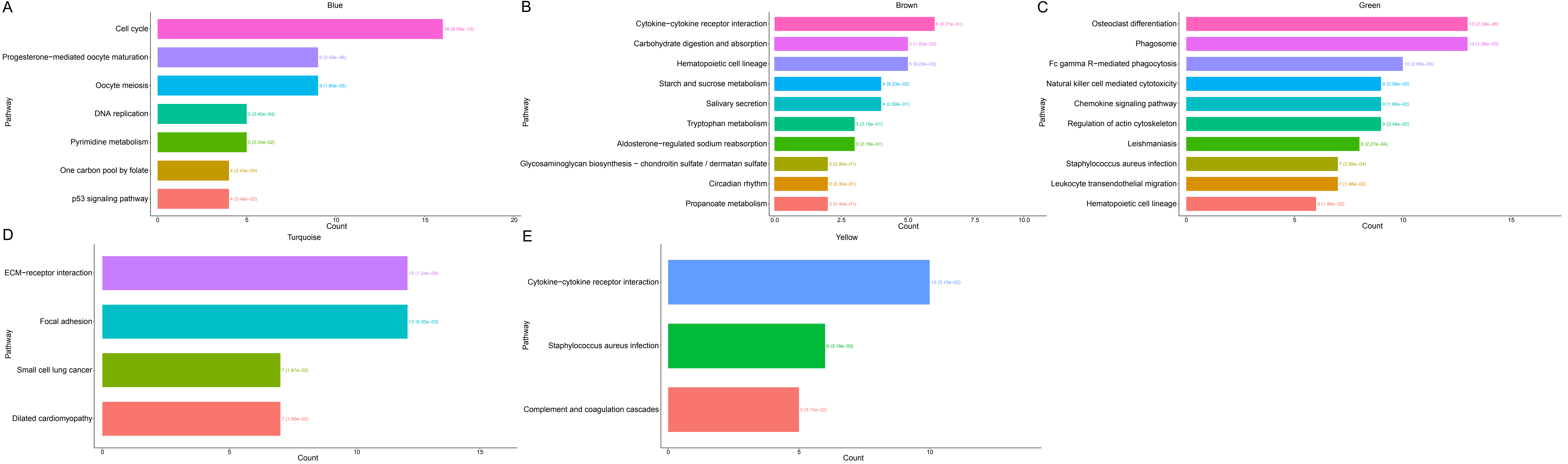

Supplement: Supplementary file 5 [file DataSheet4.ZIP › 04_Module_Gene_GO_KEGG/KEGG/Figure_6.pdf]

Blue

Pathway

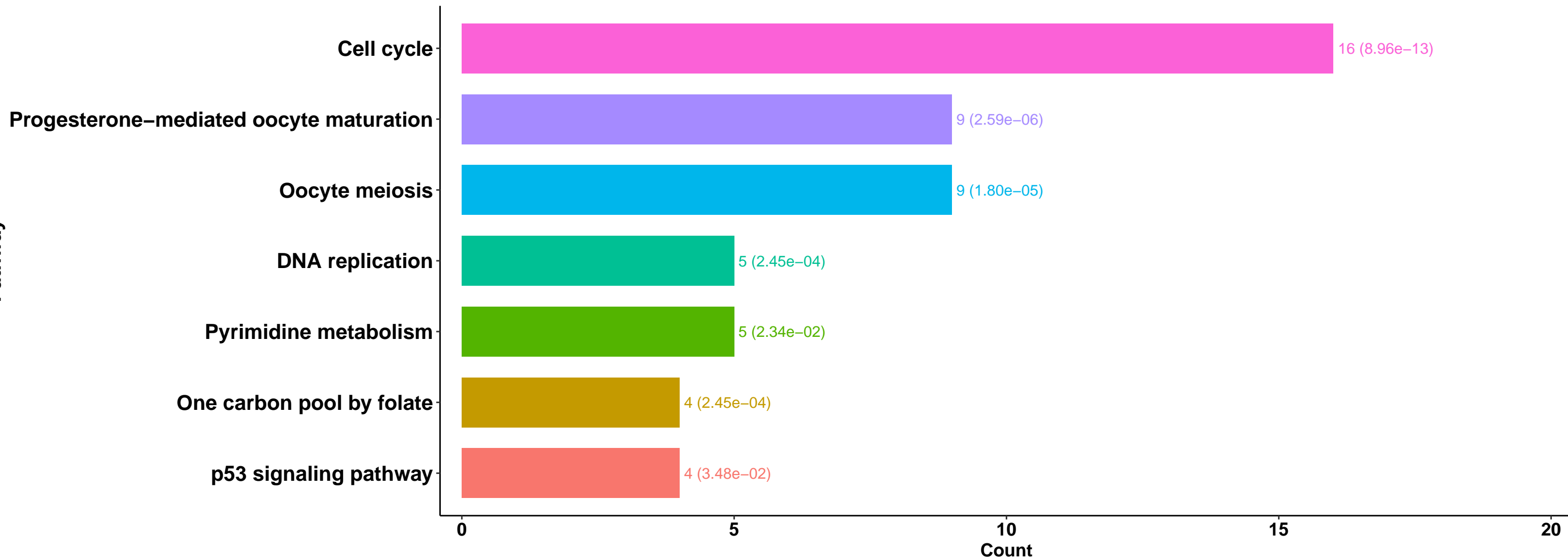

Supplement: Supplementary file 5 [file DataSheet4.ZIP › 04_Module_Gene_GO_KEGG/KEGG/blue_KEGG.pdf]

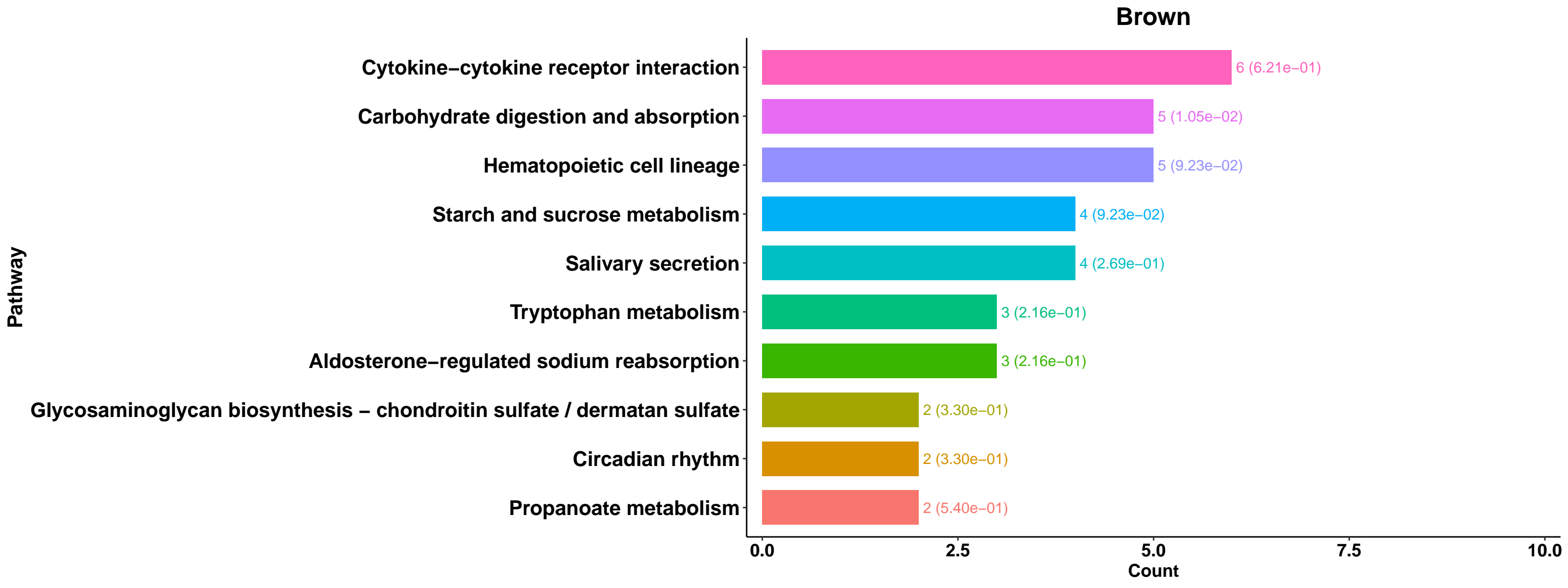

Supplement: Supplementary file 5 [file DataSheet4.ZIP › 04_Module_Gene_GO_KEGG/KEGG/brown_KEGG.pdf]

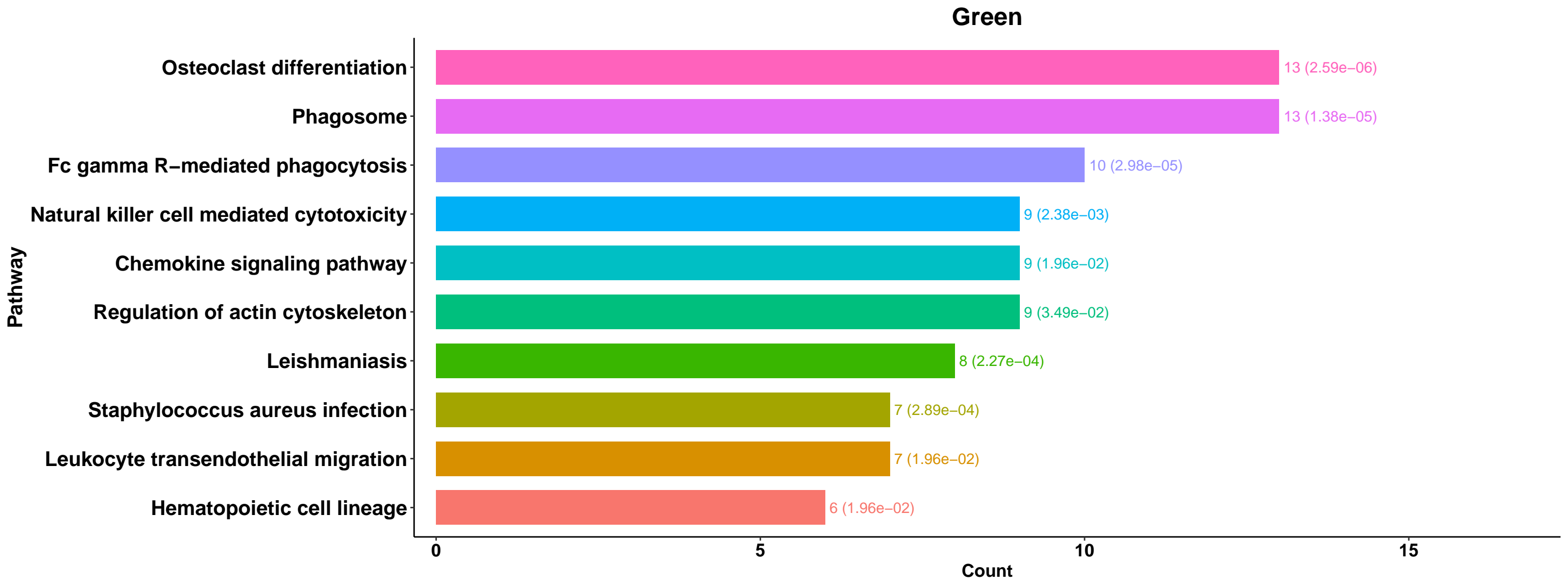

Supplement: Supplementary file 5 [file DataSheet4.ZIP › 04_Module_Gene_GO_KEGG/KEGG/green_KEGG.pdf]

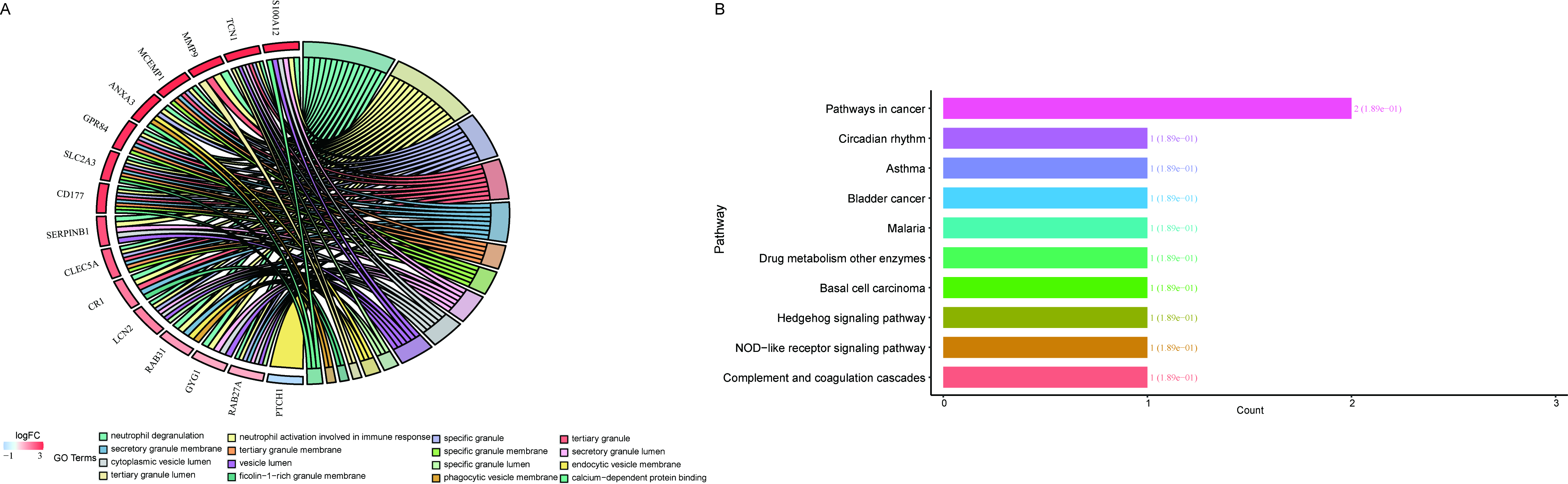

Supplement: Supplementary file 7 [file Image1.TIF]

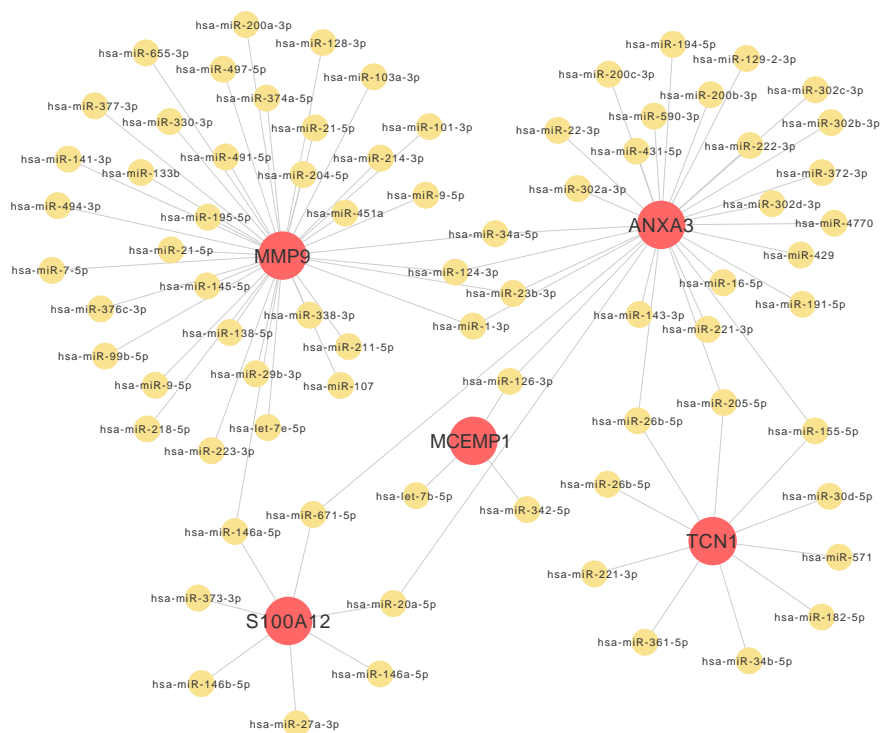

Supplement: Supplementary file 8 [file DataSheet10.ZIP › 10_Five_Gene_miRNA_Network_Analysis/Gene_miRNA_network.pdf]

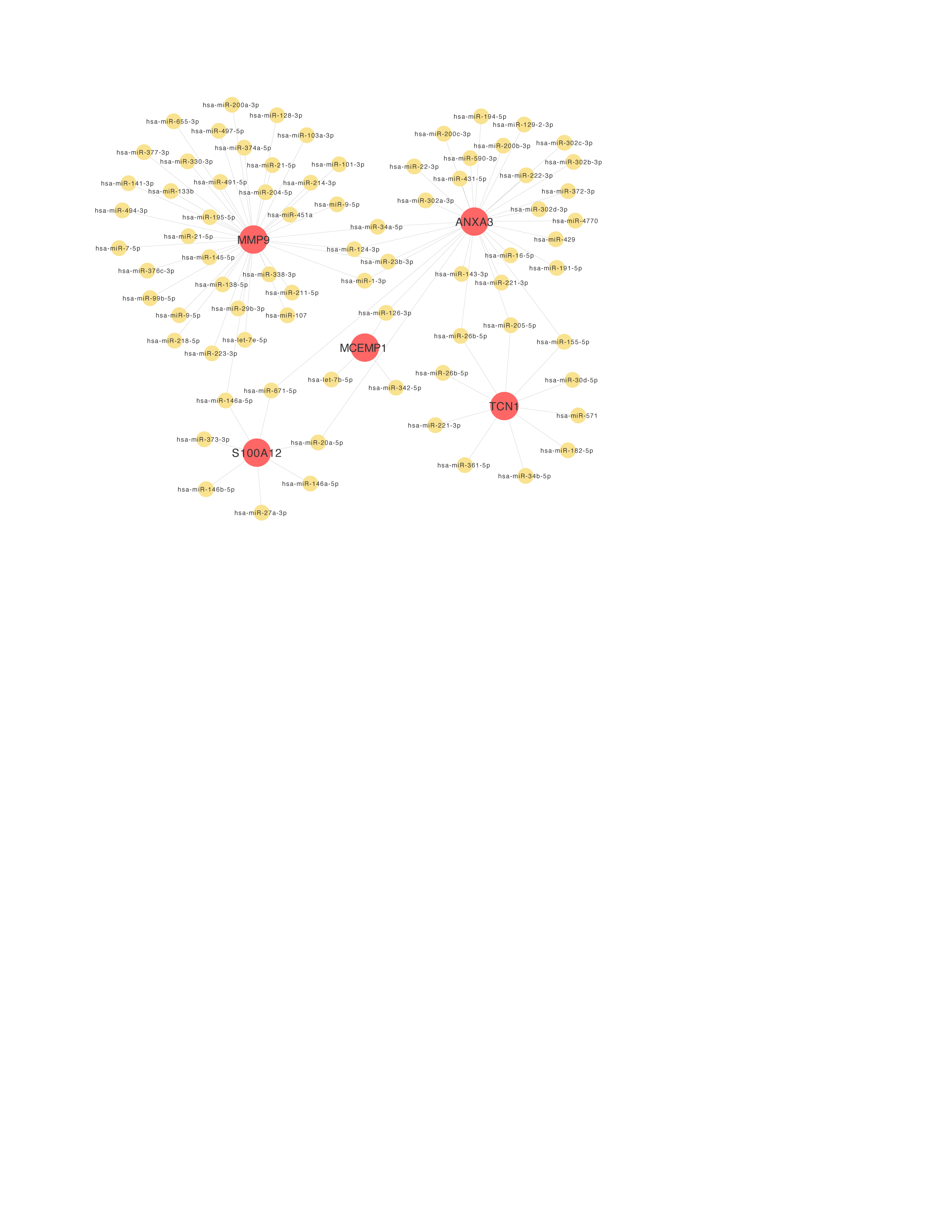

Supplement: Supplementary file 8 [file DataSheet10.ZIP › 10_Five_Gene_miRNA_Network_Analysis/Gene_miRNA_network.tif]

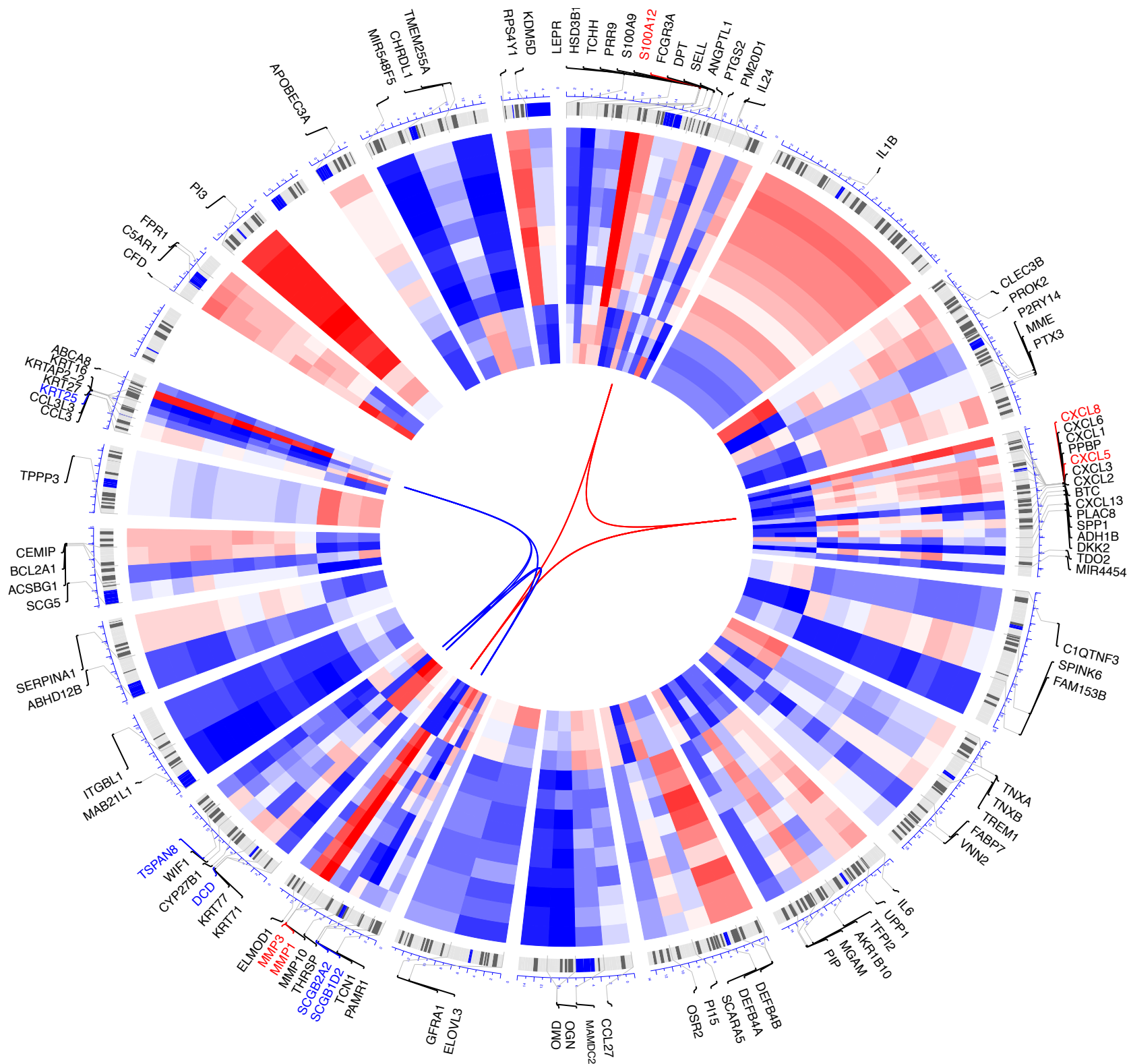

Supplement: Supplementary file 10 [file DataSheet12.ZIP › 02_Diff_Enrichment_Annalysis/01_Diff_Analysis/Circos.pdf]

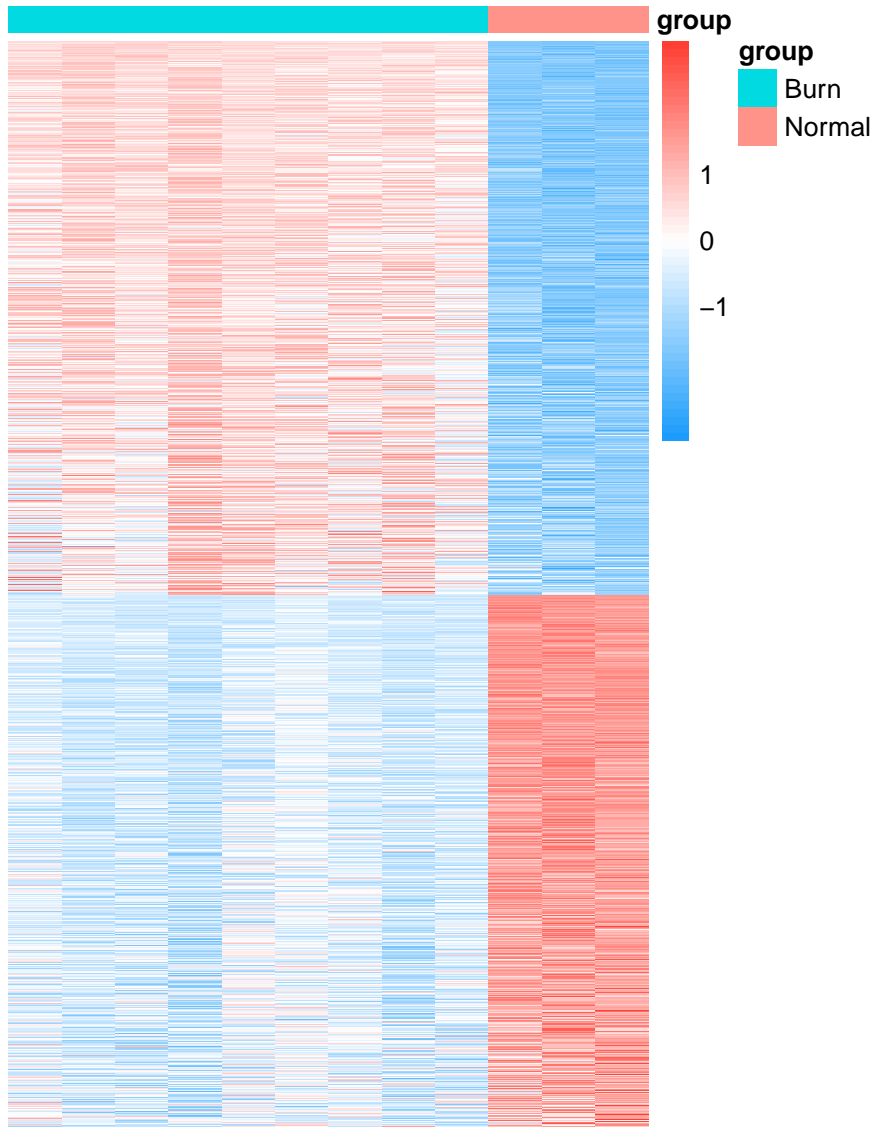

Supplement: Supplementary file 10 [file DataSheet12.ZIP › 02_Diff_Enrichment_Annalysis/01_Diff_Analysis/Heatmap.pdf]

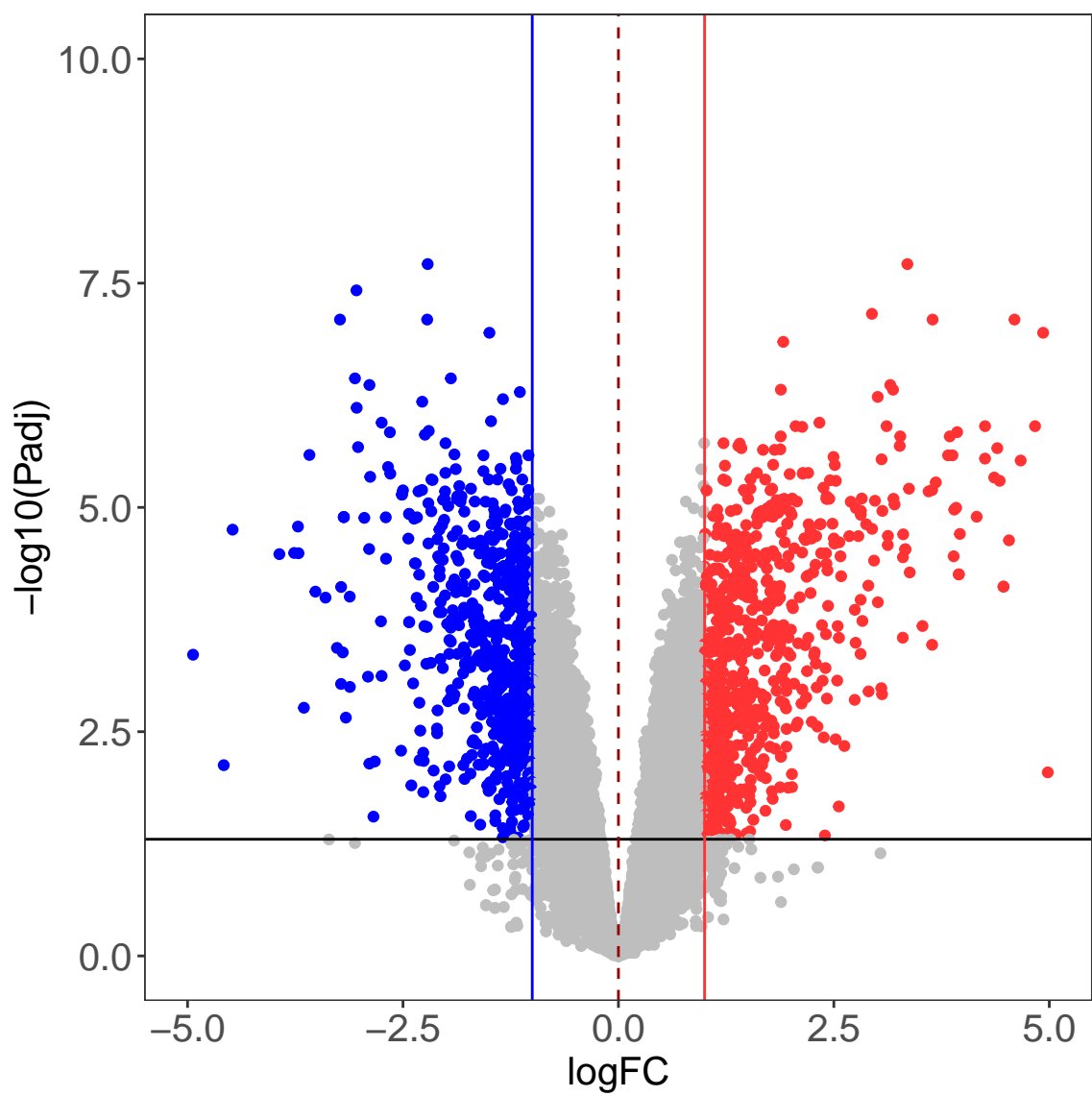

Supplement: Supplementary file 10 [file DataSheet12.ZIP › 02_Diff_Enrichment_Annalysis/01_Diff_Analysis/Volcano.pdf]

# The Most Enriched GO Terms

GO Terms

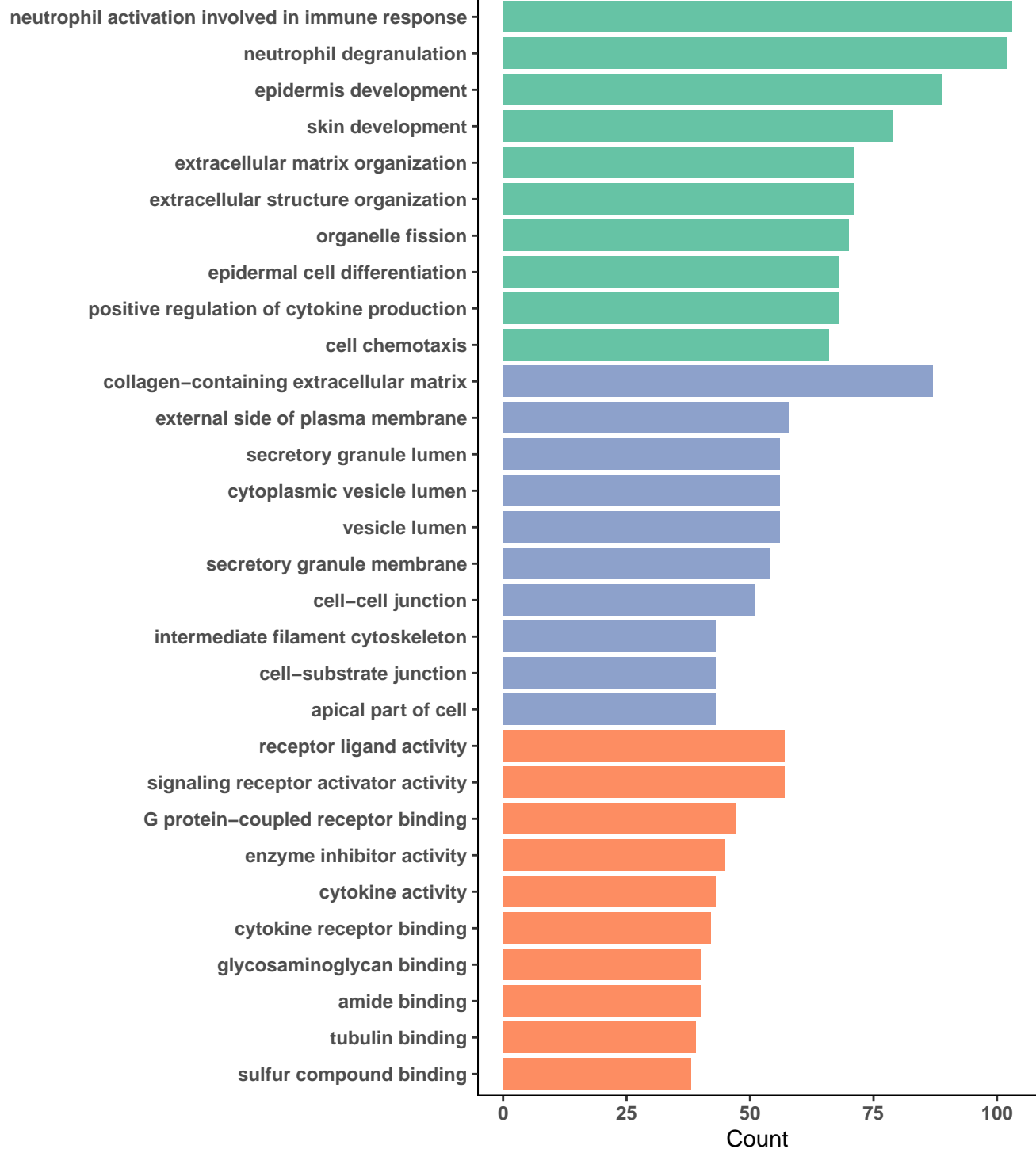

Ontology

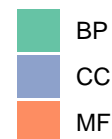

Supplement: Supplementary file 10 [file DataSheet12.ZIP › 02_Diff_Enrichment_Annalysis/02_GO/DEGs_GO.pdf]

The Enriched KEGG Pathways

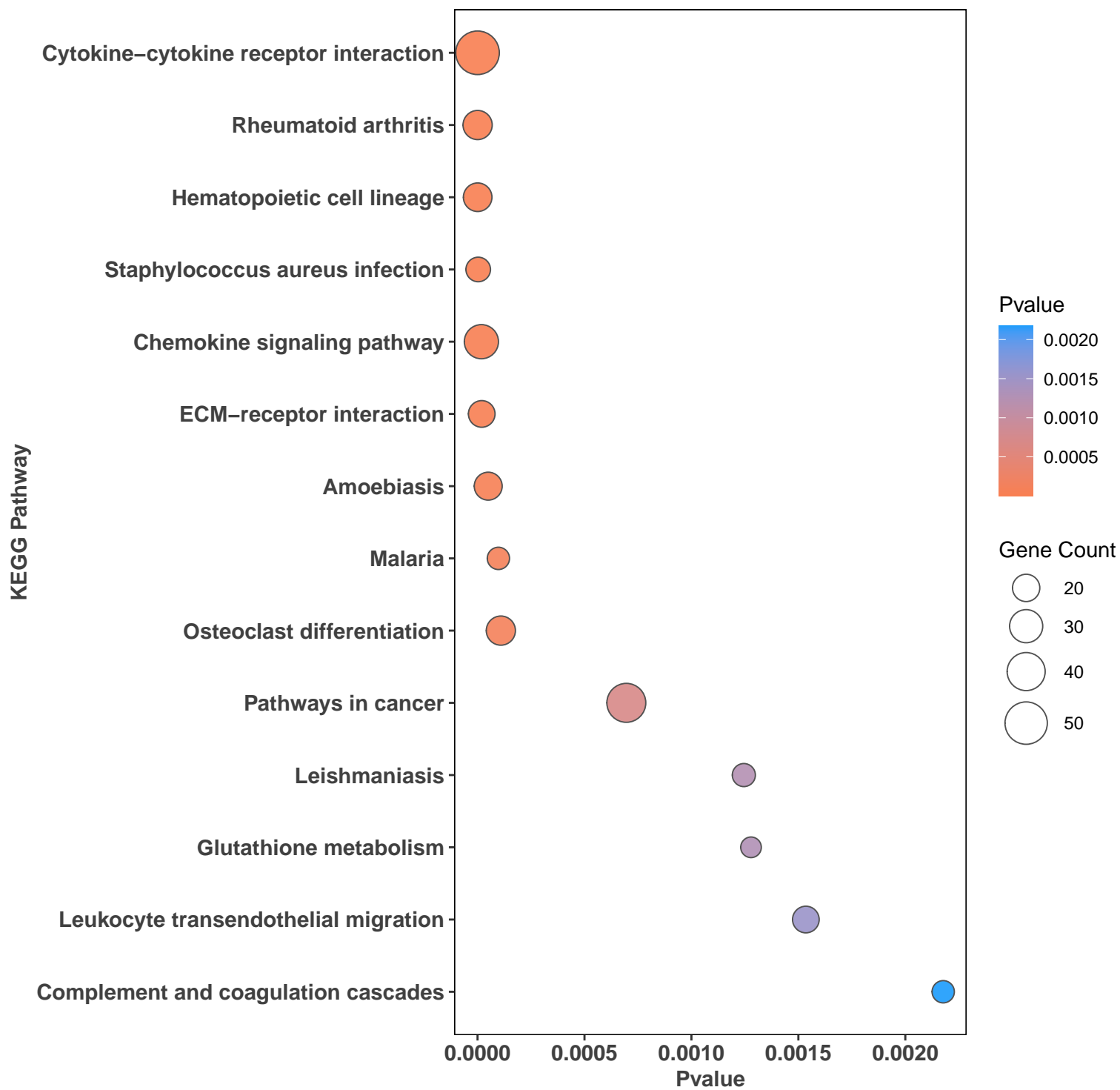

Supplement: Supplementary file 10 [file DataSheet12.ZIP › 02_Diff_Enrichment_Annalysis/03_KEGG/DEGs_KEGG.pdf]

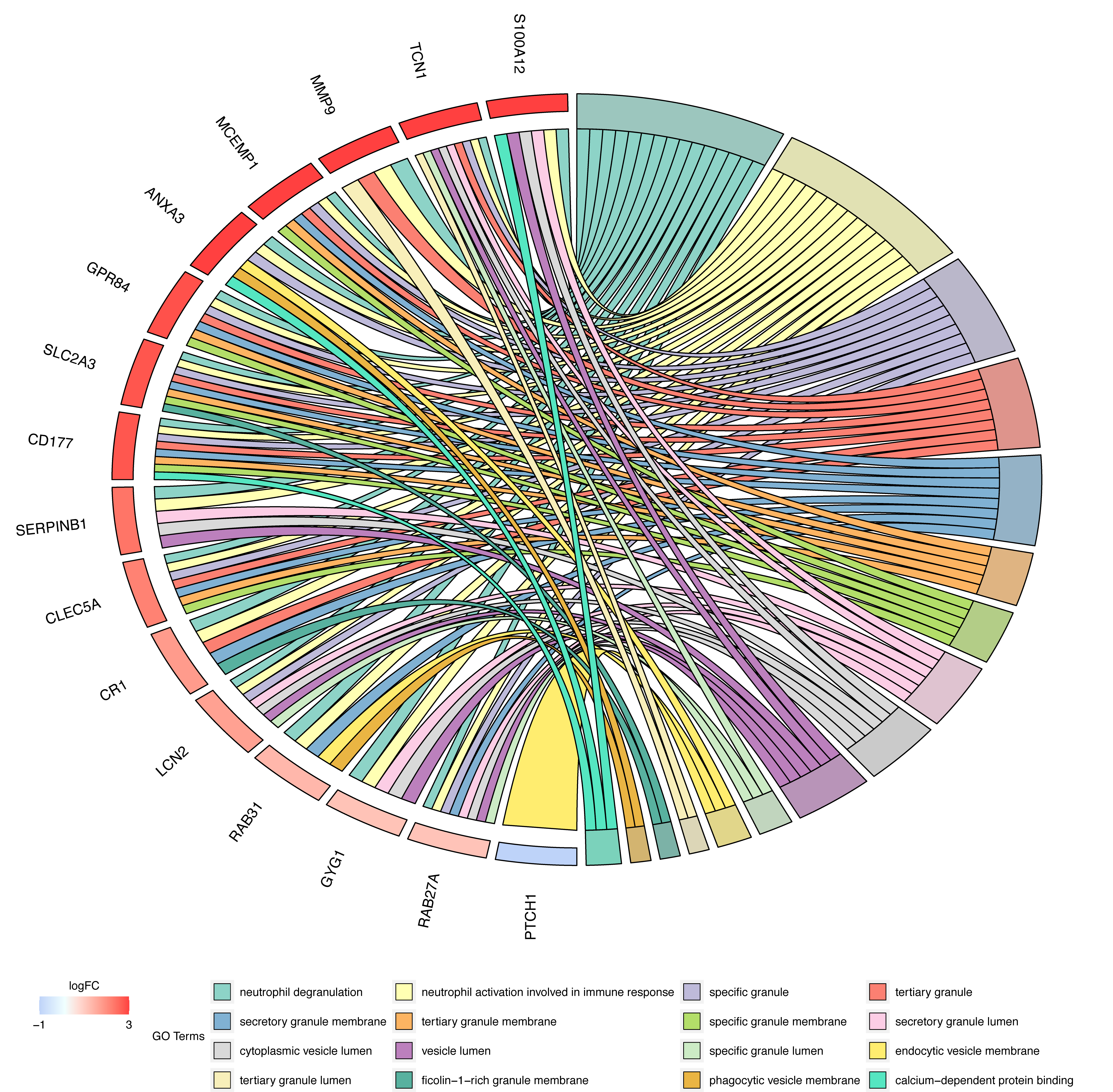

Supplement: Supplementary file 12 [file DataSheet5.ZIP › 05_Hub_Genes_Enrichment_Analysis/01_Yellow_Module_GO.tif]

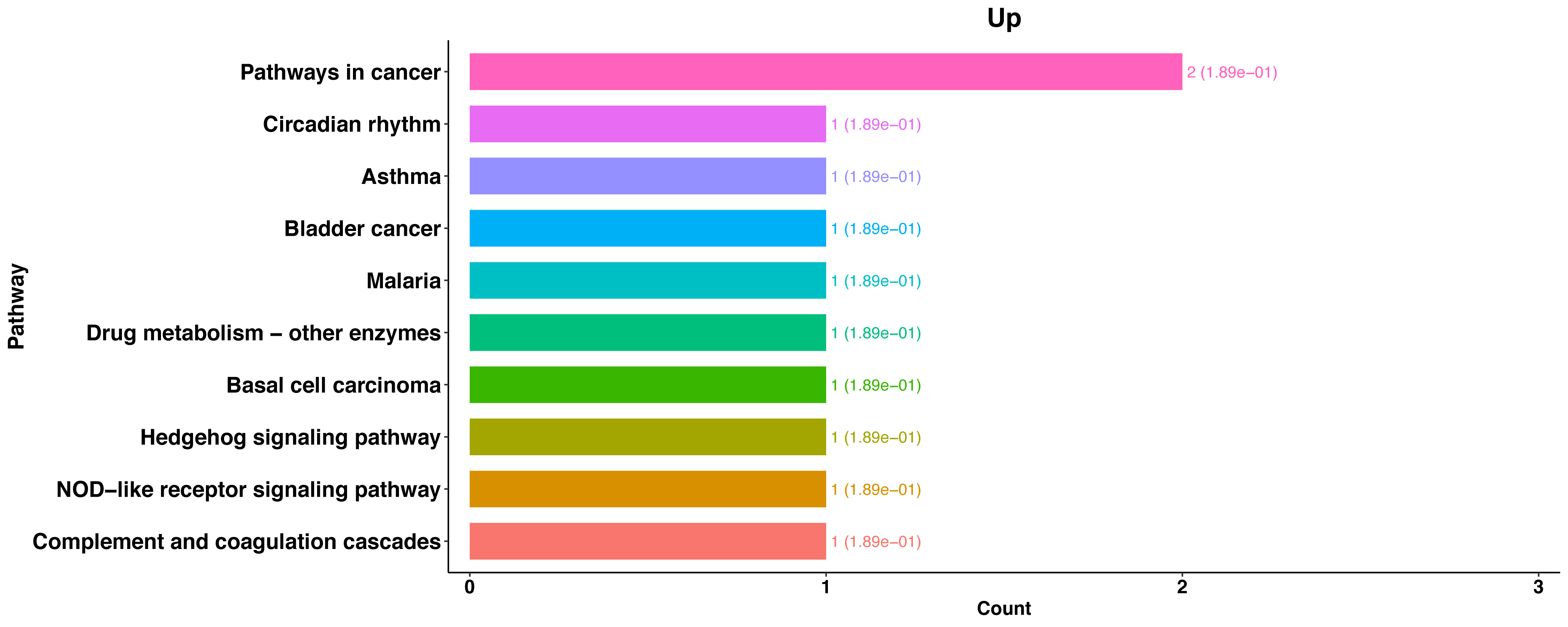

Supplement: Supplementary file 12 [file DataSheet5.ZIP › 05_Hub_Genes_Enrichment_Analysis/02_Yellow_Module_KEGG.tif]

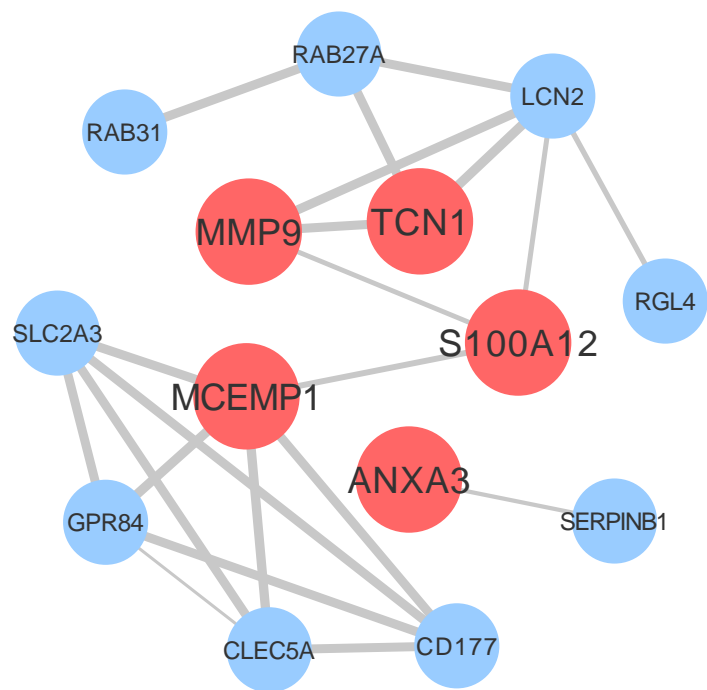

Supplement: Supplementary file 13 [file DataSheet7.ZIP › 06_Hub_Genes_PPI/Yellow_Module_Hub_Genes_PPI.pdf]

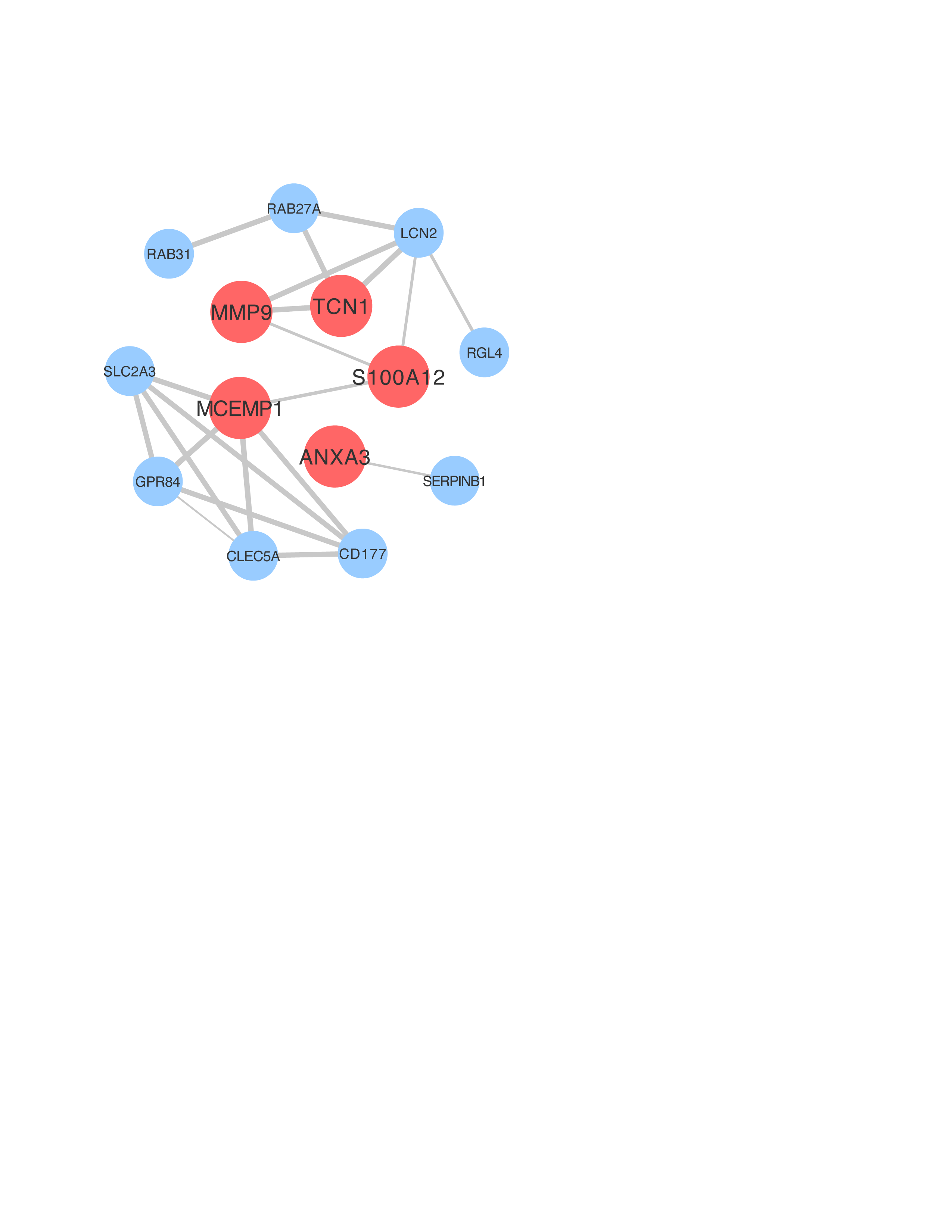

Supplement: Supplementary file 13 [file DataSheet7.ZIP › 06_Hub_Genes_PPI/Yellow_Module_Hub_Genes_PPI.tif]
