# Supplementary material for: Identification and Verification of Five Potential Biomarkers Related to Skin and Thermal Injury Using Weighted Gene Co-Expression Network Analysis
Source: Front Genet. 2022 Jan 3;12:781589. doi: 10.3389/fgene.2021.781589 (PMC8762241; doi:10.3389/fgene.2021.781589)
Supplement: Supplementary file 5 [file DataSheet4.ZIP › 04_Module_Gene_GO_KEGG/GO/blue_GO.pdf]

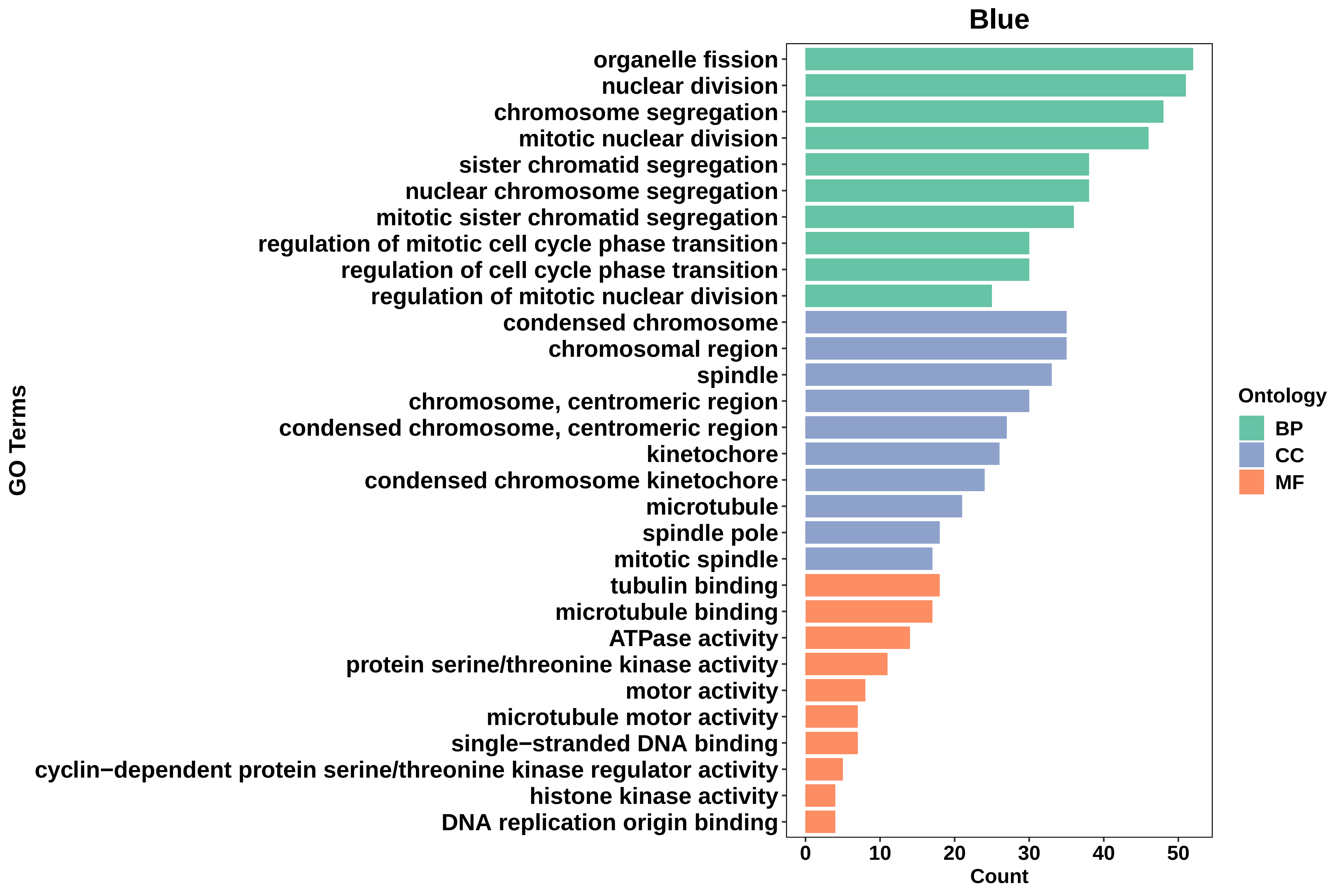
Blue
organelle fission
nuclear division
chromosome segregation
mitotic nuclear division
sister chromatid segregation
nuclear chromosome segregation
mitotic sister chromatid segregation
regulation of mitotic cell cycle phase transition
regulation of cell cycle phase transition
regulation of mitotic nuclear division
condensed chromosome
chromosomal region
spindle
chromosome, centromeric region
condensed chromosome, centromeric region
kinetochore
condensed chromosome kinetochore
microtubule
spindle pole
mitotic spindle
tubulin binding
microtubule binding
ATPase activity
protein serine/threonine kinase activity
motor activity
microtubule motor activity
single−stranded DNA binding
cyclin−dependent protein serine/threonine kinase regulator activity
histone kinase activity
DNA replication origin binding
GO Terms
0
10
20
30
40
50
Count
Ontology
BP
CC
MF
