# Supplementary material for: Identification and Verification of Five Potential Biomarkers Related to Skin and Thermal Injury Using Weighted Gene Co-Expression Network Analysis
Source: Front Genet. 2022 Jan 3;12:781589. doi: 10.3389/fgene.2021.781589 (PMC8762241; doi:10.3389/fgene.2021.781589)
Supplement: Supplementary file 5 [file DataSheet4.ZIP › 04_Module_Gene_GO_KEGG/GO/green_GO.pdf]

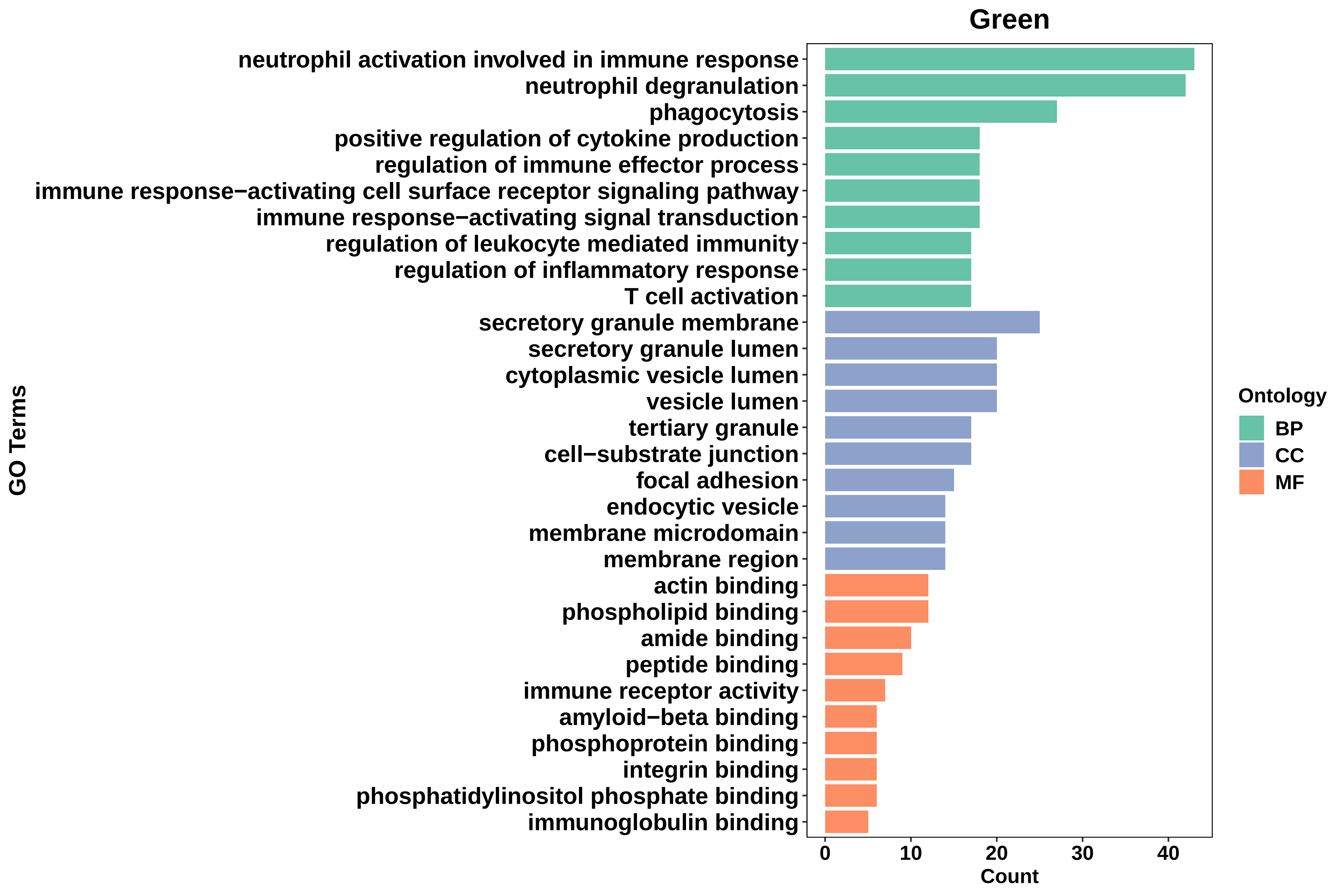
Green
GO Terms
neutrophil activation involved in immune response
neutrophil degranulation
phagocytosis
positive regulation of cytokine production
regulation of immune effector process
immune response−activating cell surface receptor signaling pathway
immune response−activating signal transduction
regulation of leukocyte mediated immunity
regulation of inflammatory response
T cell activation
secretory granule membrane
secretory granule lumen
cytoplasmic vesicle lumen
vesicle lumen
tertiary granule
cell−substrate junction
focal adhesion
endocytic vesicle
membrane microdomain
membrane region
actin binding
phospholipid binding
amide binding
peptide binding
immune receptor activity
amyloid−beta binding
phosphoprotein binding
integrin binding
phosphatidylinositol phosphate binding
immunoglobulin binding
0
10
20
30
40
Count
Ontology
BP
CC
MF
