# Supplementary material for: Identification and Verification of Five Potential Biomarkers Related to Skin and Thermal Injury Using Weighted Gene Co-Expression Network Analysis
Source: Front Genet. 2022 Jan 3;12:781589. doi: 10.3389/fgene.2021.781589 (PMC8762241; doi:10.3389/fgene.2021.781589)
Supplement: Supplementary file 5 [file DataSheet4.ZIP › 04_Module_Gene_GO_KEGG/KEGG/turquoise_KEGG.pdf]

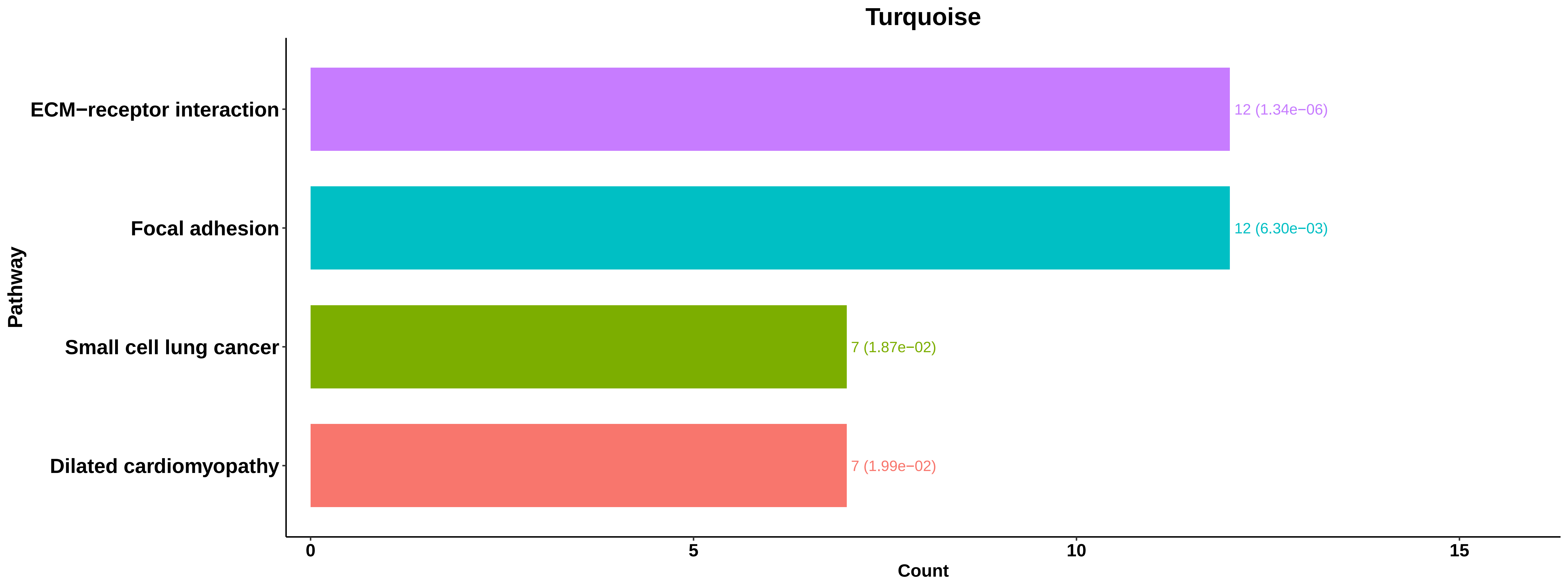
Turquoise
ECM−receptor interaction
Focal adhesion
Small cell lung cancer
Dilated cardiomyopathy
12 (1.34e−06)
12 (6.30e−03)
7 (1.87e−02)
7 (1.99e−02)
Pathway
0
5
10
15
Count
