# Supplementary material for: Identification and Verification of Five Potential Biomarkers Related to Skin and Thermal Injury Using Weighted Gene Co-Expression Network Analysis
Source: Front Genet. 2022 Jan 3;12:781589. doi: 10.3389/fgene.2021.781589 (PMC8762241; doi:10.3389/fgene.2021.781589)
Supplement: Supplementary file 5 [file DataSheet4.ZIP › 04_Module_Gene_GO_KEGG/KEGG/yellow_KEGG.pdf]

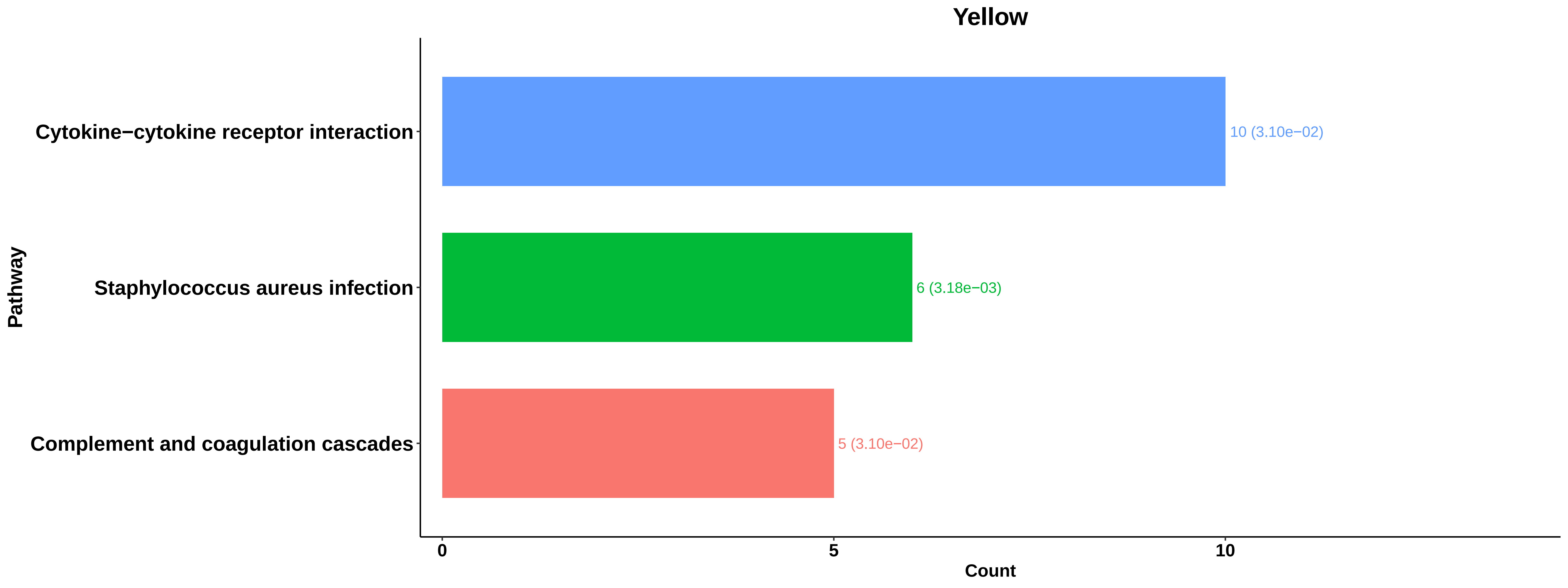

Yellow
Cytokine−cytokine receptor interaction
Staphylococcus aureus infection
Complement and coagulation cascades
10 (3.10e−02)
6 (3.18e−03)
5 (3.10e−02)
Pathway
0
5
10
Count
