# Supplementary material for: Identification and Verification of Five Potential Biomarkers Related to Skin and Thermal Injury Using Weighted Gene Co-Expression Network Analysis
Source: Front Genet. 2022 Jan 3;12:781589. doi: 10.3389/fgene.2021.781589 (PMC8762241; doi:10.3389/fgene.2021.781589)
Supplement: Supplementary file 12 [file DataSheet5.ZIP › 05_Hub_Genes_Enrichment_Analysis/01_Yellow_Module_GO.pdf]

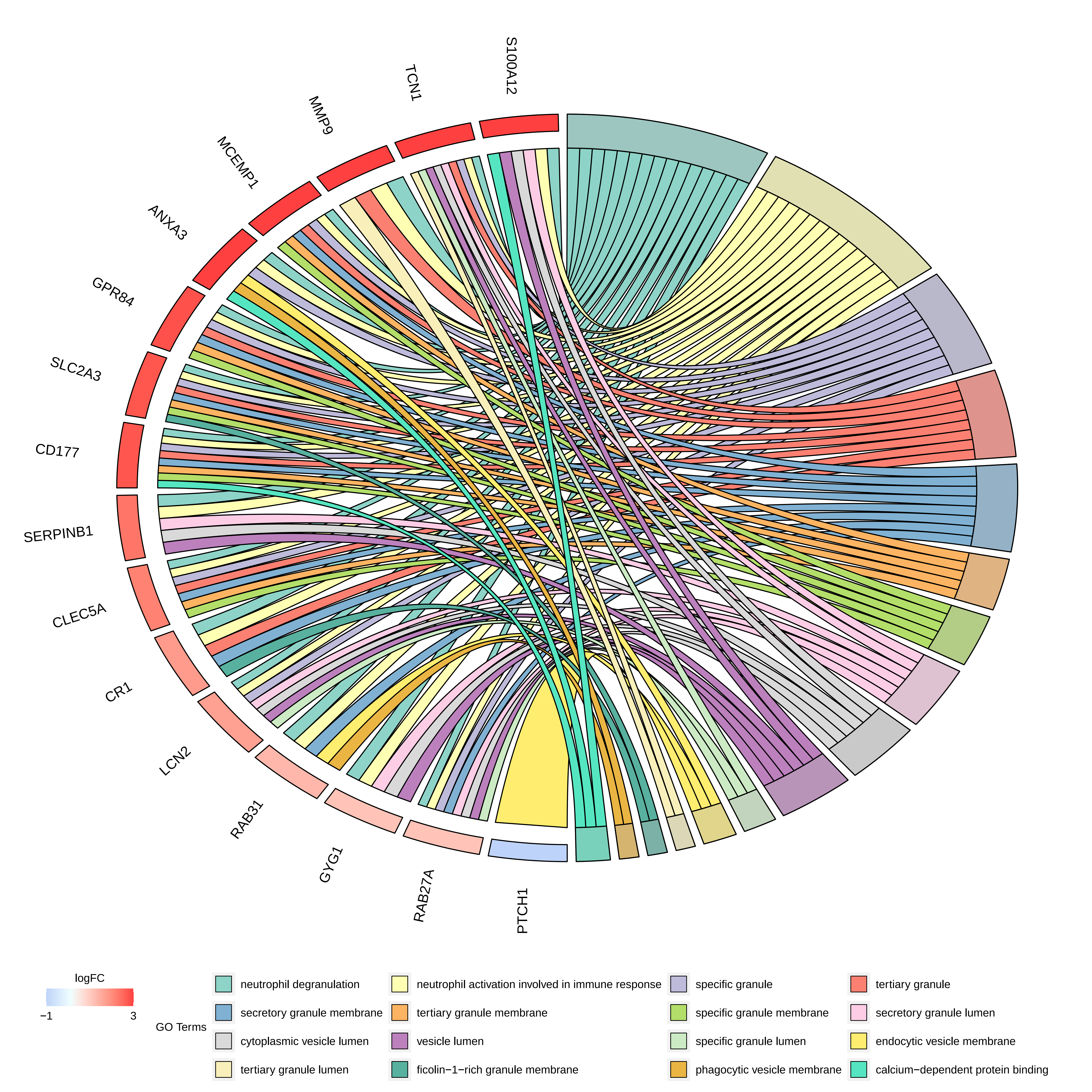
S100A12
TCN1
MMP9
MCEMP1
ANXA3
GPR84
SLC2A3
CD177
SERPINB1
CLEC5A
CR1
LCN2
RAB31
GYG1
RAB27A
PTCH1
logFC
−1
3
GO Terms
neutrophil degranulation
neutrophil activation involved in immune response
specific granule
tertiary granule
secretory granule membrane
tertiary granule membrane
specific granule membrane
secretory granule lumen
cytoplasmic vesicle lumen
vesicle lumen
specific granule lumen
endocytic vesicle membrane
tertiary granule lumen
ficolin−1−rich granule membrane
phagocytic vesicle membrane
calcium−dependent protein binding
