# Supplementary material for: Identification and Verification of Five Potential Biomarkers Related to Skin and Thermal Injury Using Weighted Gene Co-Expression Network Analysis
Source: Front Genet. 2022 Jan 3;12:781589. doi: 10.3389/fgene.2021.781589 (PMC8762241; doi:10.3389/fgene.2021.781589)
Supplement: Supplementary file 12 [file DataSheet5.ZIP › 05_Hub_Genes_Enrichment_Analysis/02_Yellow_Module_KEGG.pdf]

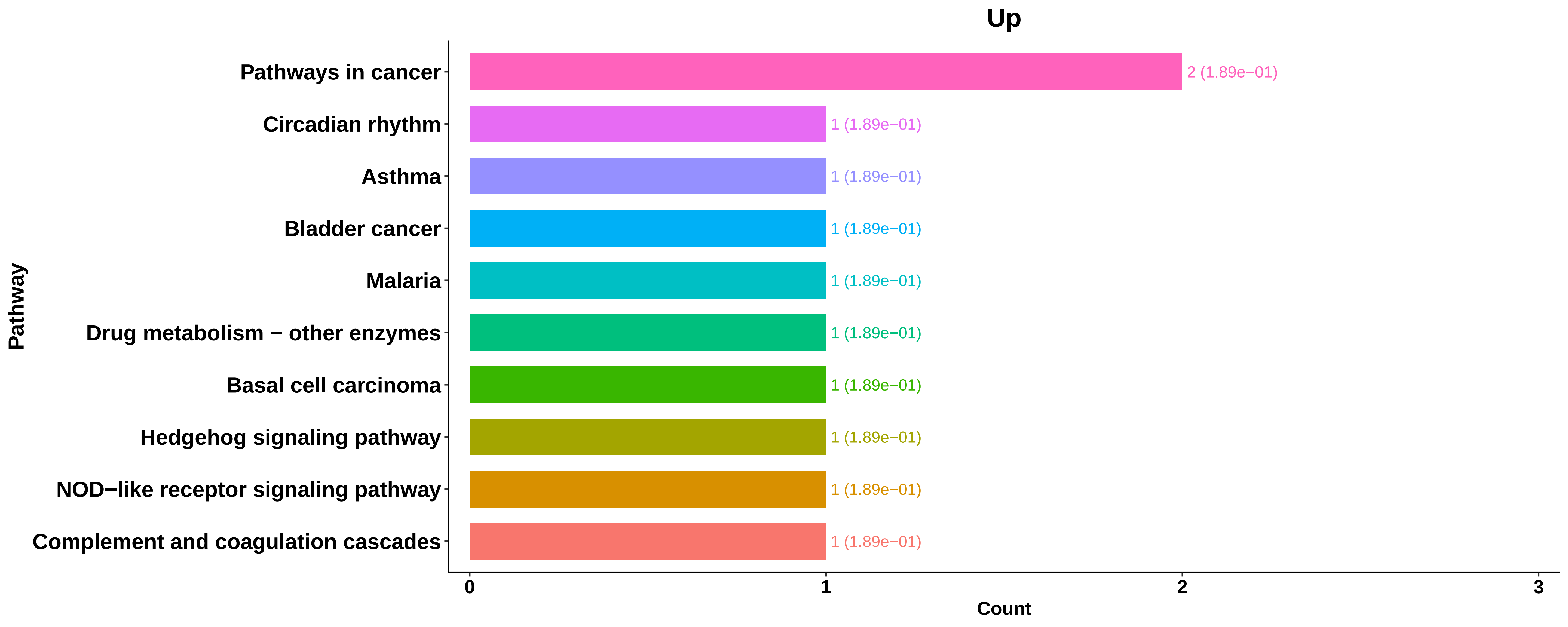
Up
Pathway
Pathways in cancer
Circadian rhythm
Asthma
Bladder cancer
Malaria
Drug metabolism − other enzymes
Basal cell carcinoma
Hedgehog signaling pathway
NOD−like receptor signaling pathway
Complement and coagulation cascades
2 (1.89e−01)
1 (1.89e−01)
1 (1.89e−01)
1 (1.89e−01)
1 (1.89e−01)
1 (1.89e−01)
1 (1.89e−01)
1 (1.89e−01)
1 (1.89e−01)
1 (1.89e−01)
0
1
2
3
Count
